# ﻿Four new species of mygalomorph spiders (Araneae, Halonoproctidae and Theraphosidae) from the Colombian Pacific region (Bahía Solano, Chocó)

**DOI:** 10.3897/zookeys.1166.101069

**Published:** 2023-06-06

**Authors:** Mariana Echeverri, Sebastián Gómez Torres, Nicolás Pinel, Carlos Perafán

**Affiliations:** 1 Área de Sistemas Naturales y Sostenibilidad, Universidad EAFIT, Medellín, Colombia Universidad EAFIT Medellín Colombia; 2 Facultad de Ciencias Exactas y Naturales, Universidad de Caldas, Manizales, Colombia Universidad de Caldas Manizales Colombia

**Keywords:** Chocó Biogeographic Region, *Euthycaelus*, Mecana, *Melloina*, *Neischnocolus*, tarantula, trapdoor spider, Tumbes-Chocó-Magdalena hotspot, *Ummidia*

## Abstract

The Colombian Pacific coast is an amazing natural region, immersed in one of the most unknown biodiversity hotspots in the world. An expedition carried out in the north of this area, at the Jardín Botánico del Pacífico (JBP) in Bahía Solano, Chocó, focused on studying the diversity of the mygalomorph spider fauna, allowed us to discover four new species included in the families Halonoproctidae and Theraphosidae. The trapdoor species *Ummidiasolana***sp. nov.**, and the theraphosids species *Euthycaeluscunampia***sp. nov.** (Schismatothelinae), *Melloinapacifica***sp. nov.** (Glabropelmatinae), and *Neischnocolusmecana***sp. nov.** (Theraphosinae) are illustrated, diagnosed, and described in detail. Photographs of somatic features and copulatory organs and a distribution map are provided. Morphological, taxonomical, and biogeographical aspects are discussed for each species. All these taxonomic novelties represent the first records of these genera for the region, expanding the range of geographic distribution of each of them. This work constitutes the first effort focused on characterizing the community of Mygalomorphae species in the Chocó Biogeographic Region.

## ﻿Introduction

The enigmatic groups of Mygalomorphae spiders, known in a broad sense as tarantulas in America, constitute approximately 6% of the total number of spider species described (Bond et al. 2012). Primarily distributed in the tropics, most mygalomorph spiders remain to be discovered (Pérez-Miles and Perafán 2017; Opatova et al. 2019; Montes de Oca et al. 2022). They include hairy spiders (tarantulas sensu stricto), trapdoor spiders, funnel-web spiders, millimeter-sized spiders with little use of the silk, bald-legged spiders with the ability to attach substrate to their bodies, among other medium-sized spiders (Raven 1985; Hedin and Bond 2006; Bond et al. 2012). They are predatory animals, relatively generalists of other arthropods, most of them with terrestrial and fossorial habits, even though some groups present arboreal habits (Coyle 1986; Pérez-Miles and Perafán 2017).

While a few groups of trapdoor spiders can perform short-range ballooning (Bristowe 1939; Coyle 1983, 1985; Coyle et al. 1985; Ferretti et al. 2013; Rossi et al. 2021), mygalomorph spiders are primarily terrestrial and relatively sedentary (Raven 1980, 1985, 2010; Pérez-Miles and Perafán 2017). Consequently, many groups have restricted geographic distributions and high levels of endemism, which makes them a highly informative group for conservation studies, environmental monitoring, and biogeography research (Raven 2010; Foelix 2011; Ferretti et al. 2012, 2014; Perafán et al. 2020). In this way, many unexplored areas, especially in megadiverse regions, have a high potential for new mygalomorph species to be discovered.

The Colombian Pacific region is a fascinating area for its biological characteristics because it’s located in the hearth of the Chocó Biogeographic Region. This area constitutes a global biodiversity hotspot, the ninth most biodiverse in the world and one of the most unknown (Cano et al. 2018; Pérez-Escobar et al. 2019). The Colombian Pacific region is distinguished by its immense biodiversity, reported in numerous studies focused especially on birds, mammals, amphibians, reptiles, fishes and plants (e.g., Myers et al. 2000; Hilty and Brown 2001; Mosquera et al. 2007; Sánchez-Gárcés 2017; SIB 2022), and to a lesser extent in some groups of arthropods: myriapods, hemipterans, dipterans, hymenopterans, among others (Martínez-Torres et al. 2011; Padilla-Gil and Arcos 2011; Escovar et al. 2013; González-Córdoba and Montoya-Lerma 2014; Lopez et al. 2014; Padilla-Gil 2017; Narváez-Vásquez et al. 2021). Studies about its arachnid diversity are scarce in the literature, and those addressing Mygalomorphae altogether absent (Paz 1988; Paz and Raven 1990; Perafán 2017; Perafán and Valencia-Cuellar 2018).

**Figure 1.**
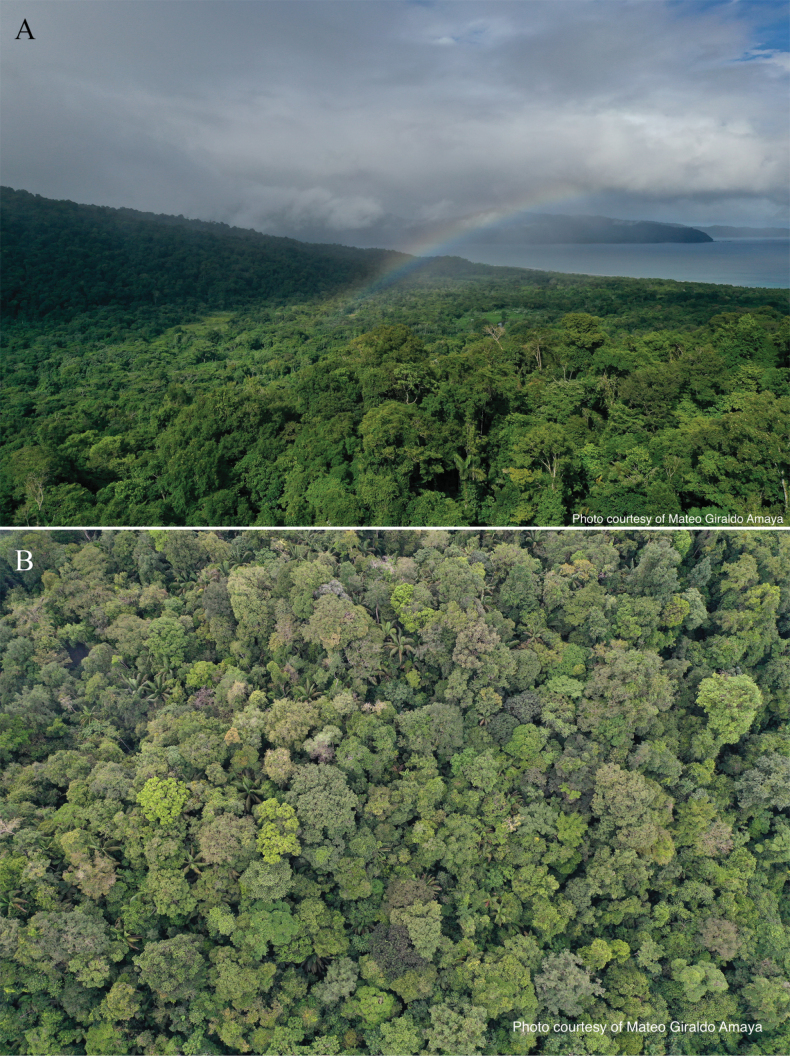
Tropical rainforest of the Jardín Botánico del Pacífico, Colombian Chocó Biogeographic Region **A** aerial view of the forest and the Pacific Ocean **B** overhead view of the forest.

Despite the great potential that Colombia has to become a worldwide reference on Mygalomorphae diversity, due to its geographical location and its enormous variety of ecosystems, only 34 of the 50 species described for the country are known by both sexes, and most of them are distributed in the Andean region (Perafán 2017; WSC 2023). This scenario highlights the need to conduct studies that complement the taxonomic gaps and the scarce information that exists in other natural regions that have been little explored (Cifuentes et al. 2016; Perafán 2017; Perafán and Valencia-Cuellar 2018; Valencia-Cuellar et al. 2019). The taxonomic novelties presented in this paper are part of the results obtained from a biological expedition carried out in the Jardín Botánico del Pacífico (JBP), focused on Mygalomorphae spiders. The JBP, located in Bahía Solano, is a tourist area and natural reserve that plays a key role in the conservation of the tropical rainforest and mangroves of the Colombian Chocó Biogeographic Region (Fig. 1).

In this first approach to the knowledge of the Mygalomorphae fauna from the rainforest of the Colombian Pacific, we illustrate, describe, and discuss one species from the Halonoproctidae trapdoor spiders, *Ummidiasolana* sp. nov., and three Theraphosidae tarantulas included in different subfamilies, as follow: *Euthycaeluscunampia* sp. nov. (Schismatothelinae), *Neischnocolusmecana* sp. nov. (Theraphosinae), and *Melloinapacifica* sp. nov (Glabropelmatinae). All these new taxonomic records represent first reports of these genera for the region, which extends the range of geographical distribution of each of them. The results of this work constitute a contribution to the knowledge of the biological diversity of one of the areas with the greatest specific richness of species and endemism in Colombia.

## ﻿Materials and methods

**Figure 2.**
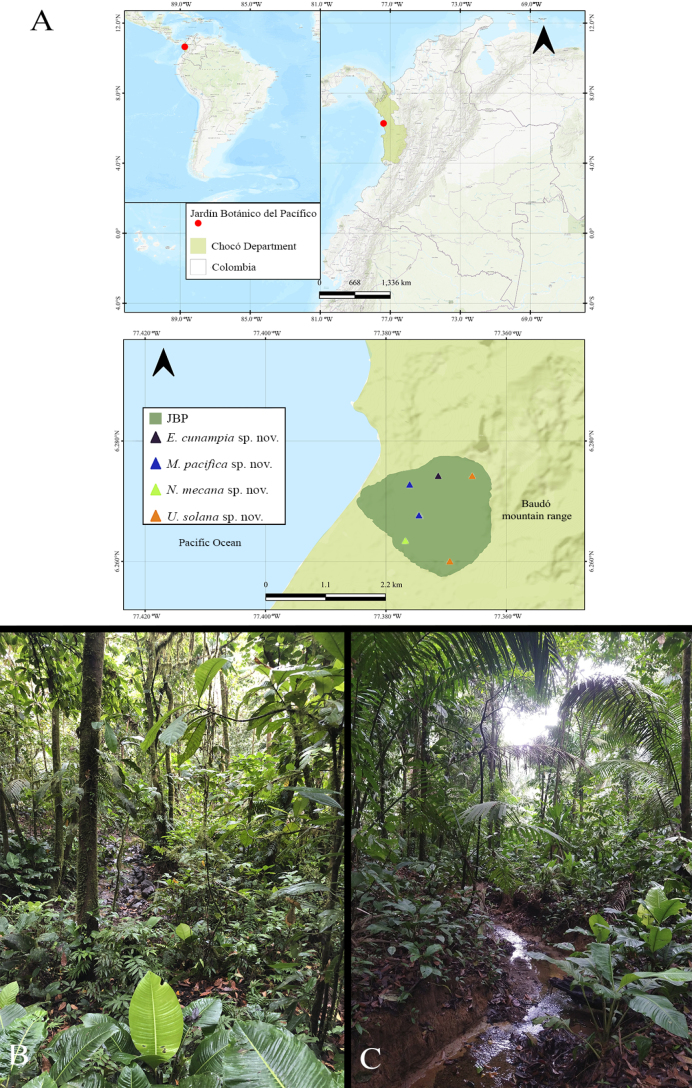
Type locality of *Euthycaeluscunampia* sp. nov, *Melloinapacifica* sp. nov., *Neischnocolusmecana* sp. nov., and *Ummidiasolana* sp. nov. **A** Geographic location of the Jardín Botánico del Pacífico (JBP), Chocó, Colombia **B, C** habitat.

All specimens herein described were collected under Universidad’s EAFIT General Collection Permit (Resolution 1566 of 24 December 2013; amended via Resolution 02493 of 31 December 2018); and deposited in the Arachnological Collection (Order Araneae) of the Instituto de Ciencias Naturales (**ICN**), Universidad Nacional de Colombia, Bogotá, Colombia, preserved in 75% ethanol. The specimens were collected during a biological expedition carried out in the Jardín Botánico del Pacífico (**JBP**), located in Bahía Solano, Chocó, Colombia (Fig. 2). The JBP has an area of 170 ha, extending from the Pacific coast to the Baudó mountain range (Fig. 2). This place is characterized by having an annual rainfall of more than 5000 mm, an average air temperature of 26 °C, and a relative humidity of 85% (Jardín Botánico del Pacífico 2014; Klinger Braham and Blandón Mosuera 2013). The field work took place during the days and nights from 10–25 February 2022.

Primary reproductive structures, palpal bulb and spermathecae, were removed for their description and photographic documentation. All photographs and descriptions of the copulatory bulb correspond to the left palp. Spermathecae were cleaned and cleared with lactic acid (85%) by immersion in a test tube and subjecting them to increased heat for short time intervals. Setae of the male tibia I and palpal tibia were removed in order to illustrate the tibial apophysis and nodules, respectively. Specimens and the structures removed were examined under a LEICA M205C stereo microscope. Photographs were taken with a stereo microscope ZEISS Stereo Discovery V12, then stacked with Helicon Focus 8.2.0 Mac OS (Helicon Soft Ltd. 2019) and processed with Adobe Photoshop CC 2022 (Adobe Inc. 2022).

All measurements are given in millimeters (mm). The total length given does not include the chelicerae or spinnerets. Eye sizes were measured as the maximum diameter in either a dorsal or frontal view and were taken with a digital micrometer. Body measurements were taken with a digital micrometer or a vernier caliper. The length and width of carapace, eye tubercle, labium and sternum are the maximum values obtained. Leg and palp measurements were taken in dorsal view along the central axis of the right-side limbs and were taken with a vernier caliper.

The general descriptive format follows Decae (2010) and Godwin and Bond (2021) for *Ummidia* and Raven (1985) and Bertani (2013) for theraphosids, with few modifications. No additional material was examined for the taxonomic analysis of any species. The diagnosis was made based on the original descriptions of its congeners and geographically related species. The diagnosis of *Ummidiasolana* sp. nov. is based on Godwin and Bond (2021), and of *Melloinapacifica* is based on Bertani (2013) and Goloboff-Szumik and Ríos-Tamayo (2022). Setae on the ventral side of the tarsus in *Ummidia* and *Melloina* are considered as pseudoscopula, according to Pérez-Miles et al. (2017). Number and disposition of spines enumerated from the anterior third to the posterior third, modified from Petrunkevitch (1925). The palpal bulb terminology used in the text and figures follows Bertani (2000) and Guadanucci and Weinmman (2014) to Theraphosinae and Schismatothelinae, respectively. Urticating setae terminology follows Cooke et al. (1972) and Kaderka et al. (2019). For conservation of these new species, the same geographic coordinates of the JBP are recorded in the original description: 6.38, -77.40; specific geographic location data are recorded in the collection label for each of them. The distribution map was produced using QGIS 3.26.1 – Buenos Aires.

Abbreviations used in the text and figures are as follows:

**A** apical keel of palpal bulb;

**ALE** anterior lateral eyes;

**AME** anterior median eyes;

**ap** apical;

**d** dorsal side;

**ITC** inferior tarsal claw;

**p** prolateral side;

**PB** prolateral branch of tibial apophysis;

**PI** prolateral inferior keel of palpal bulb;

**PLE** posterior lateral eyes;

**PME** posterior median eyes;

**PMS** posterior median spinnerets;

**PLS** posterior lateral spinnerets;

**PS** prolateral superior keel of palpal bulb;

**R** retrolateral keel of palpal bulb;

**RB** retrolateral branch of tibial apophysis;

**SLS** spine like setae;

**SR** seminal receptacles;

**STC** superior tarsal claw;

**v** ventral side.

## ﻿Taxonomy

### ﻿Family Halonoproctidae Pocock,1901

**Genus *Ummidia* Thorell, 1875**

#### ﻿ Ummidia solana sp. nov.

Taxon classificationAnimaliaAraneaeHalonoproctidae

F2736434-F432-5DE7-BC46-75498A479E49

https://zoobank.org/C21B3C70-626A-4B19-830E-25B8E74EC200

Figs 3 , 4 , 5 , 6 , 7 , 8 , 9 , 10 ; Tables 1 , 2

##### Type material.

***Holotype*** ♂: Colombia, Chocó, Bahía Solano, Jardín Botánico del Pacífico, 6.38, -77.40, elevation 60 m a.s.l., 10–25 February 2022, M. Echeverri, S. Gómez Torres and C. Perafán leg. (ICN 12356). ***Paratype*** ♀: same data as holotype, except elevation 132 m a.s.l. (ICN 12357).

##### Etymology.

The specific epithet *solana* is a noun in feminine refers to the municipality of Bahía Solano, one of the most beautiful places in the Colombian Pacific coast, recognized for having large and desolate beaches and landscapes of abundant vegetation. It is immersed in one of the world’s biodiversity hotspots. It is also said that the word “solano” means “wind from where the sun rises”.

##### Diagnosis.

**Figure 3.**
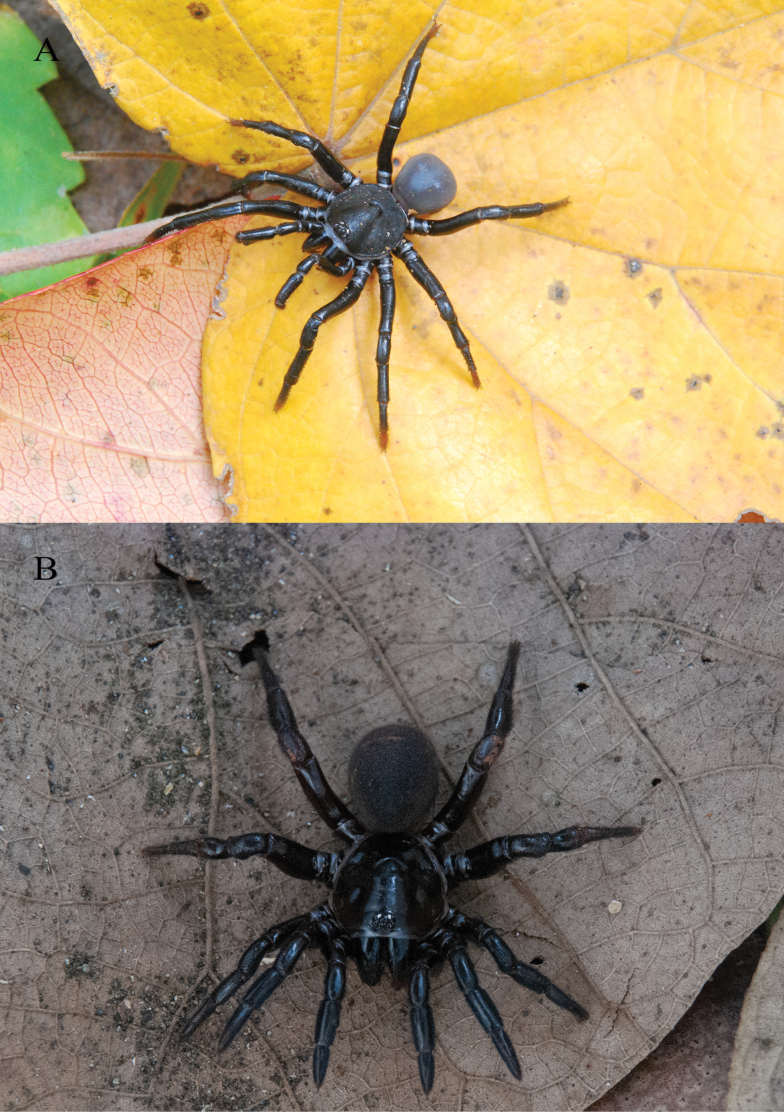
*Ummidiasolana* sp. nov., habitus **A** holotype male **B** paratype female.

*Ummidiasolana* sp. nov. can be differentiated from all geographically proximate species (see Godwin and Bond 2021) by the following combination of morphological features. Male: subcircular carapace, palpal bulb with thin and smoothly sinuous embolus, distally flattened (Fig. 7A–D); tibia I with numerous spines, 14 prolateral and 40 retrolateral; tarsus IV with defined comb on the retrolateral face (Fig. 6F) (males of *U.quijichacaca* and *U.tibacuy* unknown). Female: oval carapace, longer than wide, with strongly procurved fovea, wide and deep (Fig. 8A, B) (carapace longer than wide but angular in *U.quijichacaca* and wider than long and rounded in *U.tibacuy*, both with shallower fovea); basal segment of chelicerae with numerous lateral teeth (9–10) (4–6 in *U.quijichacaca* and *U.tibacuy*); maxillae with two sets (proximal and distal) of few cuspules of similar number (ca. 12) (Fig. 9F) (27 proximal / 24 distal in *U.quijichacaca* and 38 proximal / 15 distal in *U.tibacuy*); palp trochanter with distinct group of elongated cuspules (Fig. 9E) (unknown in the other species); labium with few weak subconical cuspules (4) (Fig. 9D) (7 in *U.quijichacaca* and 13 in *U.tibacuy*); tarsus IV with comb of long spinules on the retrolateral face (Fig. 10E) (similar in *U.quijichacaca* and alternating long and short hairs in *U.tibacuy*); and seminal receptacles straight oriented inwardly, mushroom-shaped distally subspherical, with wide rounded bulbs (Fig. 8E) (seminal receptacles mushroom-shaped but flat bulbs in *U.quijichacaca* and straight not mushroom-shaped in *U.tibacuy*). Additionally, female and male (alive) with black carapace and legs, grayish abdomen, and male with reddish brown tarsi (Fig. 3A).

##### Distribution.

Known only from the type locality (Figs 1, 2).

##### Description.

**Figure 4.**
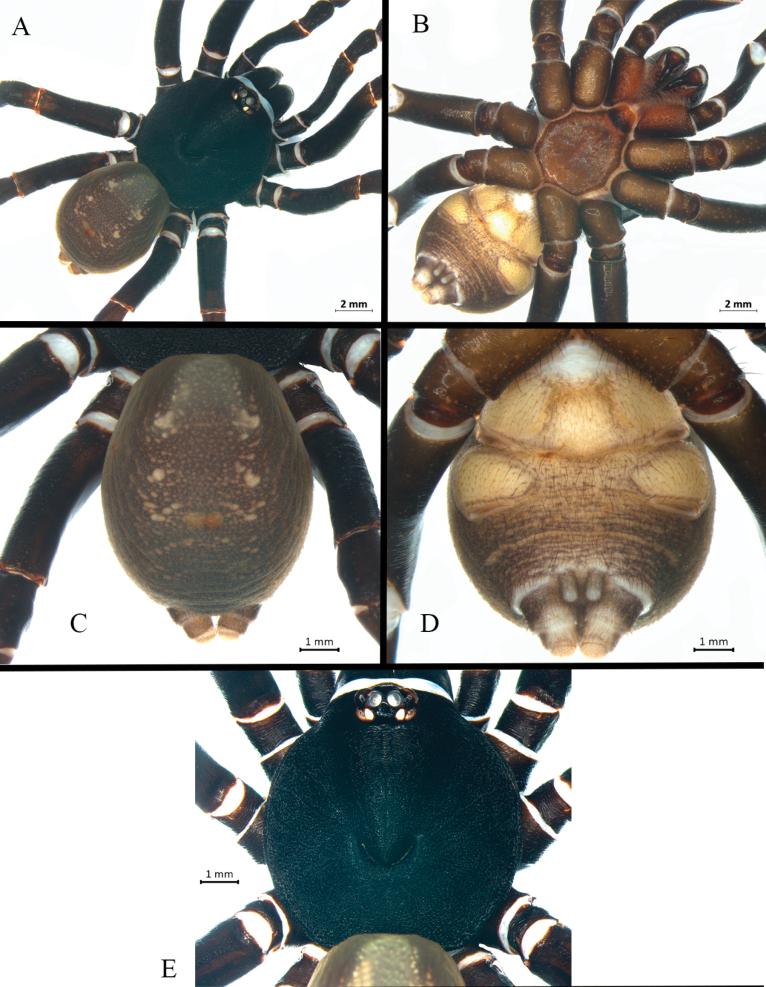
*Ummidiasolana* sp. nov., holotype male, habitus **A** dorsal view **B** ventral view **C, D** abdomen **C** dorsal view **D** ventral view **E** carapace.

**Figure 5.**
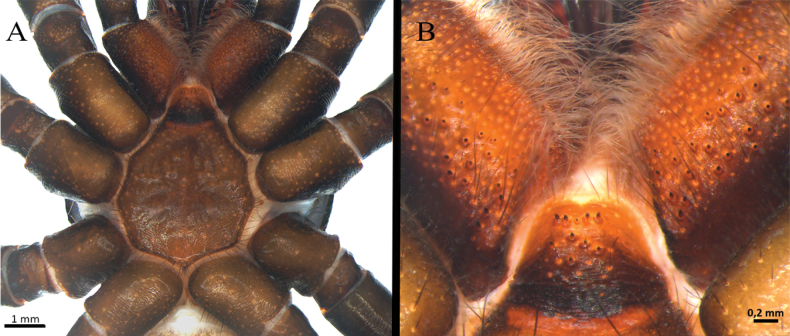
*Ummidiasolana* sp. nov., holotype male **A** sternum and coxae **B** labium and maxillae.

**Figure 6.**
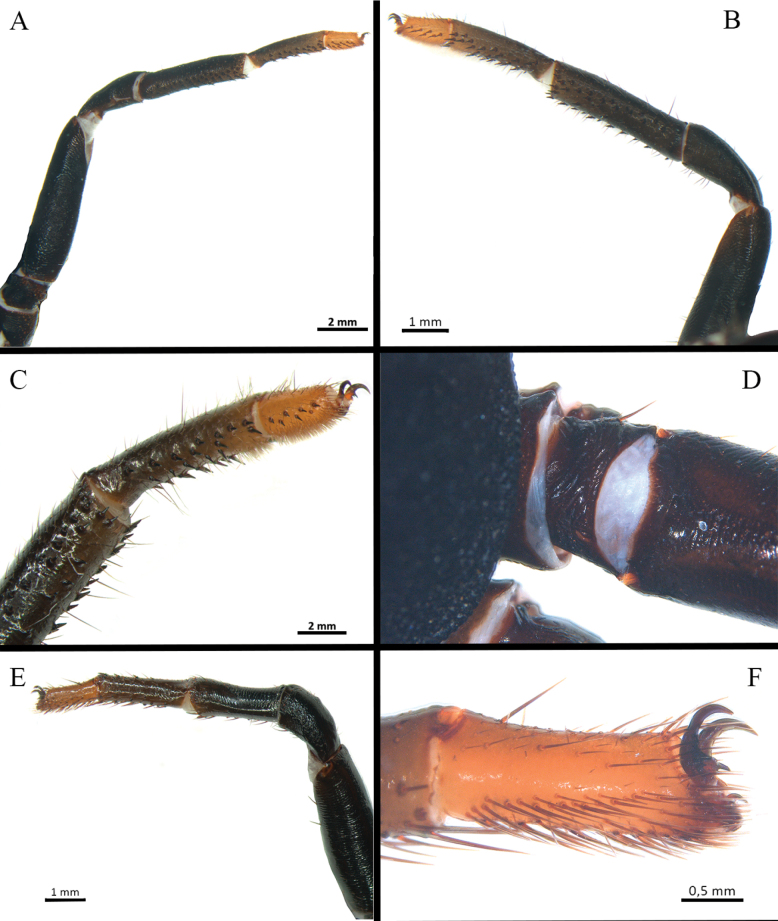
*Ummidiasolana* sp. nov., holotype male **A–C** leg I **A** retrolateral view **B** prolateral view **C** detail of lateral fields of short curvy spines **D, E** leg III **D** trochanter, blunt pointed apophysis **E** tibia III, retrolateral dorsal view, saddle-like depression **F** tarsus IV, retrolateral view, comb.

**Figure 7.**
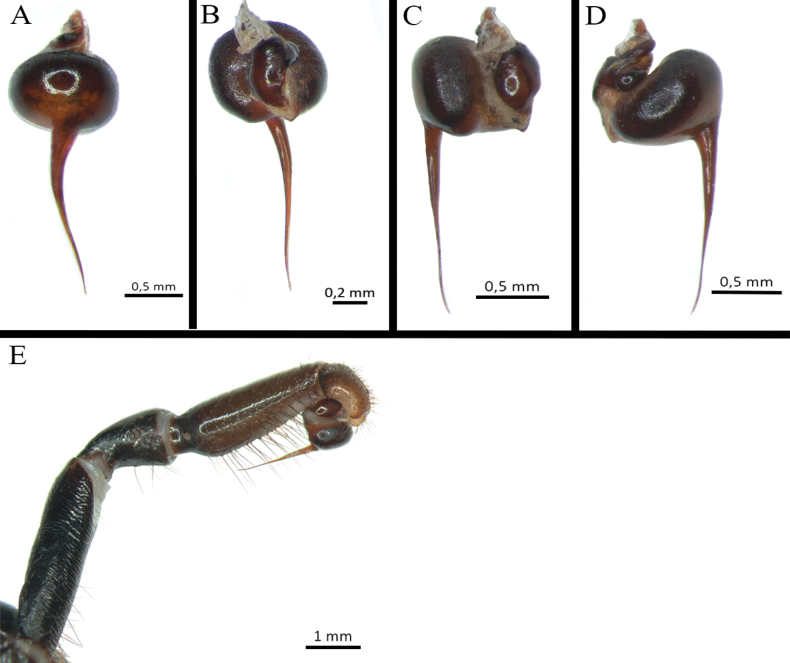
*Ummidiasolana* sp. nov., holotype male, pedipalp **A–D** copulatory bulb **A** ventral view **B** dorsal view **C** prolateral view **D** retrolateral view **E** retrolateral view.

**Male** (holotype) (Figs 3A, 4–7). Total length: 13.40. Chelicerae basal segment: length 1.37, width 1.47. Carapace: subcircular, glabrous, rugose, length 6.85, width 6.87; cephalic area elevated, length 4.8, height 0.59. Abdomen: egg-shaped with evenly distributed bristles set in strongly developed wart-like sockets. Spinnerets: PLS with three segments, total length 1.25 (basal 0.66, middle 0.29, apical digitiform 0.3); basal segments with fine, small, and macro-spigots, distal segment with numerous fine spigots and few macro-spigots. PMS with one segment, length 0.65, with numerous small spigots. (Fig. 4).

Clypeus: length 0.38, without bristles; protracted onto membranous connection between carapace and chelicerae. Eye group (Fig. 4E): eight eyes on a raised ovoid tubercle, arranged in two rows on the near anterior edge of carapace; anterior eye row procurved, posterior eye row straight. Ocular tubercle: length 0.93, width 1.33. Eye diameters and interdistances: AME 0.36 (circular), ALE 0.42 (oval), PME 0.19 (circular), PLE 0.29 (oval), AME-AME 0.09, AME-ALE 0.09, ALE-ALE 0.94, PME-PME 0.49, PME-PLE 0.04, PLE-PLE 0.97, AME-PME 0.05, ALE-PLE 0.10. Thoracic fovea (Fig. 4E): transverse, highly procurved, deep, width 2.1; 4.48 from the anterior edge of carapace. Chelicerae basal segment: furrow with ca. six prolateral / eight retrolateral teeth. Rastellum: absent. Fang long. Maxillae (Fig. 5): sub-rectangular, with ca. 24 left / 30 right cuspules uniformly distributed on the ventral posterior area; cuspules on the anterior inner edge absent. Labium (Fig. 5): semi-dome shape, length 0.95, width 1.24, with ten weak subconical cuspules. Labio-sternal junction: narrow. Palp trochanter: without cuspules. Sternum (Fig. 5A): rounded, length 3.68, width 3.63; smooth, with few setae mainly on the edge. Lacking lateral sigilla, posterior sigilla large, central, and indistinct.

Legs pattern: IV>I>II>III. Lengths of legs and palpal segments on Table 1. Tarsal claws: STC with single large and acute proximal tooth, ITC very short and steeply curved in all tarsi. Claw tufts: absent. Pseudoscopulae: tarsi I and II present, III and IV absent; metatarsi I and II present on distal edge, III and IV absent. Tarsal trichobothria: filiform present, 1–3 clavate trichobothria in all tarsi. All femora with wide membranous slits on proximal side. All legs and palp with many spiniform setae (Fig. 6). All femora and the palp without spines.

**Table 1.** *Ummidiasolana* sp. nov. Male holotype. Lengths of legs and palpal segments.

	I	II	III	IV	Palp
Femur	6.44	4.97	4.43	5.92	4.09
Patella	3.15	2.71	2.93	2.94	2.01
Tibia	4.13	3.11	2.28	3.65	3.13
Metatarsus	3.24	2.65	2.63	4.18	-
Tarsus	1.31	1.08	1.62	1.75	0.59
Total	18.27	14.52	13.89	18.44	9.82

Legs I (Fig. 6A–C) and II: lateral fields of short curvy spines on tarsus, metatarsus, tibia, and patella. Leg III (Fig. 6D, E): trochanter with blunt pointed apophysis on prolateral dorsal (Fig. 6D); femur swollen; patella strong, with prolateral field of eight short spines on half distal side; tibia short, strong saddle, flanked on either side by narrow membranous slits, with field of short spines on distal dorsal-prolateral side; metatarsus short, with dorsal field of four short spines on distal edge; tarsus short, with prolateral and retrolateral long spine field along full length of segment. Leg IV: retrolateral face of tarsus with defined comb over length of the segment (Fig. 6F).

Palp (Fig. 7): femur distally wider, tibia swollen (Fig. 7E); palpal bulb pyriform, with subtegulum small, embolus thin, smoothly sinuous, distally flattened (Fig. 7A–D).

***Coloration*.** Living spider: carapace black, rugose; ocular area black, PME yellow; chelicerae basal segment, palp, and legs black; tarsi reddish brown; abdomen gray, with cream color spotted pattern. In alcohol: carapace black; sternum brown; labium and maxillae reddish brown; legs dark brown; abdomen gray with spotted pattern; genital area, book lung openings and spinnerets light yellow.

**Figure 8.**
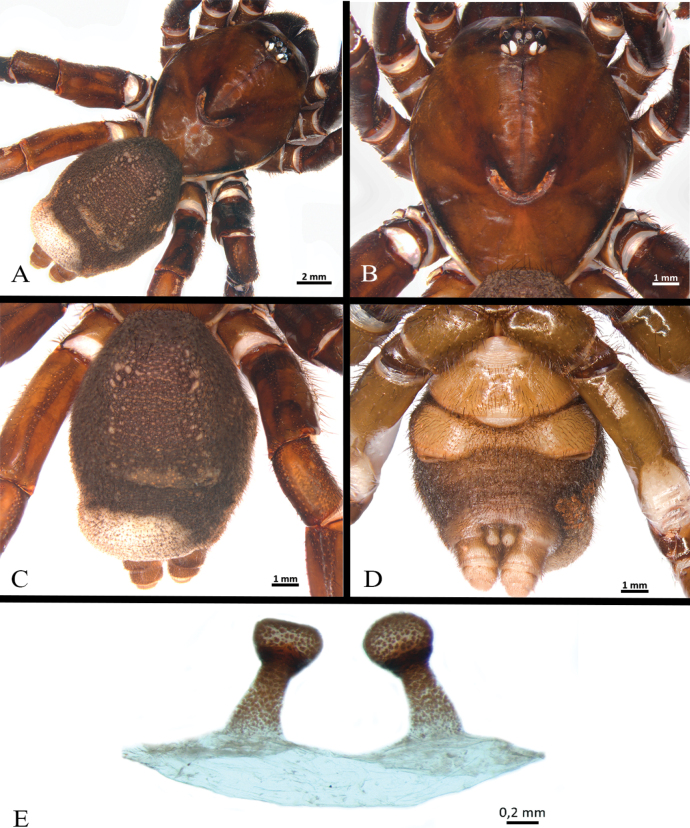
*Ummidiasolana* sp. nov., paratype female **A** habitus, dorsal view **B** carapace **C, D** abdomen **C** dorsal view **D** ventral view **E** spermathecae, ventral view.

**Figure 9.**
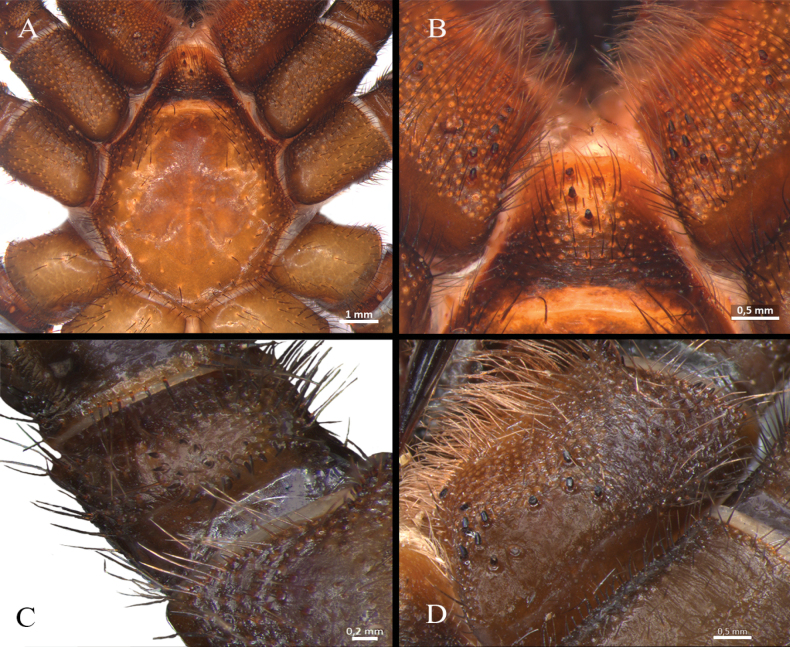
*Ummidiasolana* sp. nov., paratype female **A, B** chelicerae, rastellum **A** ventral view **B** dorsal view **C** sternum and coxae **D** labium and maxillae **E** palp trochanter **F** maxilla.

**Figure 10.**
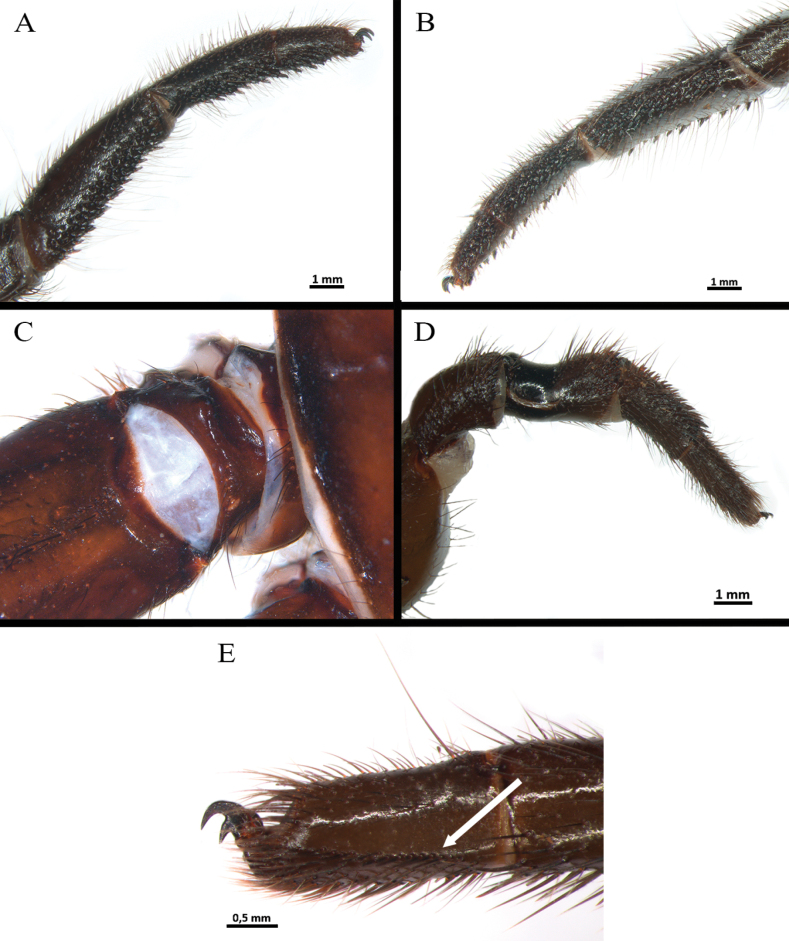
*Ummidiasolana* sp. nov., paratype female **A, B** leg I **A** retrolateral view **B** prolateral view **C, D** leg III **C** trochanter, blunt pointed apophysis **D** tibia, saddle-like depression **E** tarsus IV, retrolateral view, comb.

**Female** (paratype) (Figs 3B, 8–10). Total length: 19.1. Chelicerae basal segment: length 1.2, width 1.7. Carapace: oval, glabrous, shiny, length 10, width 7.9; cephalic area elevated, length 6.62, height 0.82. Abdomen: large, egg-shaped with evenly distributed bristles set in strongly developed wart-like sockets. Spinnerets: PLS with three segments, total length 1.63 (basal 0.77, middle 0.5, apical digitiform 0.36); basal segments with fine, small, and macro-spigots, distal segment with numerous fine spigots and few macro-spigots. PMS with one segment, length 0.78, with numerous small spigots. (Fig. 8).

Clypeus: length 0.35, with few bristles; protracted onto membranous connection between carapace and chelicerae. Eye group (Fig. 8B): eight eyes on a raised ovoid tubercle, arranged in two rows on the near anterior edge of carapace; anterior eye row procurved, posterior eye row straight, slightly recurved. Ocular tubercle: length 1.12, width 1.94. Eye diameters and interdistances: AME 0.3 (circular), ALE 0.52 (oval), PME 0.5 (oval), PLE 0.42 (oval), AME-AME 0.13, AME-ALE 0.42, ALE-ALE 1.15, PME-PME 0.45, PME-PLE 0.11, PLE-PLE 1.15, AME-PME 0.17, ALE-PLE 0.27. Thoracic fovea (Fig. 8A, B): transverse, highly procurved, deep, width 2.67; 5.32 from the anterior edge of carapace. Chelicerae basal segment: furrow with ca. ten prolateral / nine retrolateral teeth. Rastellum (Fig. 9A, B): present, formed by many stout short spines, the majority arranged in very developed prolateral process. Fang long. Maxillae (Fig. 9D, F): sub-rectangular, with cuspules organized in two groups; one group with ca. 12 strong and larger cuspules, proximal and scattered throughout most of the article; the other one with ca. 13 smaller distal cuspules, the majority occupying the distal edge. Palp trochanter (Fig. 9E): with distinct group of ca. 13 elongated cuspules. Labium (Fig. 9C, D): semi-dome shape, length 1.35, width 1.83, with four weak subconical cuspules. Labio-sternal junction: narrow. Sternum (Fig. 9C): rounded, length 5.3, width 5.33; smooth, with few setae mainly on the edge. Lacking lateral sigilla, posterior sigilla large, central, and indistinct.

Legs pattern: IV>I>III>II. Lengths of legs and palpal segments on Table 2. Tarsal claws:

**Table 2.** *Ummidiasolana* sp. nov. Female paratype. Lengths of legs and palpal segments.

	I	II	III	IV	Palp
Femur	5.25	4.77	4.59	5.96	4.70
Patella	3.57	3.36	3.07	3.46	3.24
Tibia	3.39	2.90	2.56	3.76	3.53
Metatarsus	2.63	2.34	2.45	3.79	-
Tarsus	1.21	1.70	2.51	2.32	2.67
Total	16.05	15.07	15.18	19.29	14.14

STC with single large and acute proximal tooth, ITC very short and steeply curved in all tarsi. Claw tufts: absent. Pseudoscopulae: absent in all legs. Tarsal trichobothria: Palpal tarsus with ca. 13 clavate trichobothria on medial edge and four filiform trichobothria on distal edge; tarsi I–IV with 1–3 clavate trichobothria and few filiform trichobothria. All femora with wide membranous slits on distal side. All legs and palp with many spiniform setae (Fig. 10). All legs femora and patellae I and II without spines.

Legs I (Fig. 10A, B) and II, and palp. Palp: femur with a wide row of fine spine-like setae (SLS) along 60% of the segment; patella with a low promedial lobe and four short and wide spines; tibia with a prolateral and retrolateral wide row of curved spines along full length of segment; metatarsus-tarsus with a prolateral and retrolateral wide row of curved spines along full length of segment. Femora I and II: with central rows of short fine SLS along full length of segment. Patellae I and II: with a low retromedial lobe, with three dorsal central rows of fine and short SLS along full length of segment, with some ventral long SLS. Tibiae I and II: with several row of dorsal SLS; tibia I with a wide prolateral and retrolateral row of curved spines along full length of segment; tibia II with a ventral row of lightly curved spines along full length of segment, with a prolateral row of short, curved spines along full length of segment. Metatarsi I and II: with dorsal SLS along full length of segment, and a prolateral and retrolateral row of curved spines along full length of segment. Tarsi I and II: with dorsal SLS and a prolateral and retrolateral row of short, curved spines.

Leg III (Fig. 10C, D). Trochanter: with blunt pointed apophysis on prolateral dorsal (Fig. 10C). Femur: swollen, with dorsal and ventral rows of SLS. Patella: dorsal with a central row of SLS along full length of segment, with ca. 13 prodorsal short and strong spines in distal edge. Tibia (Fig. 10D): short, strong saddle, flanked on either side by narrow membranous slits on either side, field of short and fine spines in median side, field of short spines on distal prolateral and dorsal side, with a dorsal row of SLS along full length of segment. Metatarsus: with dorsal and prolateral strong spines, larger than tibiae spines, with a ventral row of strong spines on the distal edge of segment. Tarsus: with prolateral and retrolateral long spines and SLS along full length of the segment.

Leg IV. Trochanter and femur: unmodified. Patella: with a wide dorsal central row, prolateral fields of short spines, rise in size toward distal side. Tibia: swollen, dorsal and prolateral with a row of short and fine spines along full length of segment. Metatarsus and tarsus: prodorsal and retrodorsal with SLS along full length of segment, and with ventral long spines covering the totality of the tarsus and 80% of the metatarsus. Retrolateral face of tarsus with defined comb of long spinules over length of the segment (Fig. 10E).

Spermathecae (Fig. 8E): two seminal receptacles, straight, oriented inwardly, mushroom-shaped distally subspherical, granulated appearance; proximal part tubular, glandular, medial part formed by a sclerotized band, distal part subspherical, glandular.

***Coloration*.** Living spider: carapace black, smooth, shiny, darker than male; ocular area black, PME yellow; chelicerae basal segment, palp, and legs black; abdomen dark gray, with cream color spotted pattern. In alcohol: carapace dark brown; legs and palp brown with darker overtones, mainly in femora and in the distal segments of all legs; sternum, labium, and maxillae brown; abdomen greyish brown with spotted pattern; genital area, book lung openings, and spinnerets light yellow.

##### Remarks.

*Ummidiasolana* sp. nov. is the third species described from the genus and the family Halonoproctidae for Colombia. Godwin and Bond (2021) previously described the species *Ummidiaquijichacaca* and *Ummidiatibacuy*, both distributed in the center of the country, in the Eastern Cordillera of the Andean Region, and known only from female specimens. *U.solana* sp. nov. broadens the geographical distribution of the genus since it represents the first record from the Chocó Biogeographical Region. The male was captured walking at night while the female was captured inside her cave, also active at night. Her burrow was built on the ground under leaf litter.

### ﻿Family Theraphosidae Thorell, 1869

**Subfamily Glabropelmatinae**

**Genus *Melloina* Brignoli, 1985**

#### ﻿ Melloina pacifica sp. nov.

Taxon classificationAnimaliaAraneaeTheraphosidae

30200412-8EC6-55A9-B1E7-51CC3FDD526D

https://zoobank.org/8E218194-9D9E-46DE-ACD5-731D51BBA978

Figs 11 , 12 , 13 , 14 , 15 , 16 , 17 , 18 , 19 ; Tables 3 , 4

##### Type material.

***Holotype*** ♂: Colombia, Chocó, Bahía Solano, Jardín Botánico del Pacífico, 6.38, -77.40, 45 m a.s.l., 10–25 February 2022, M. Echeverri, S. Gómez Torres and C. Perafán leg. (ICN 12358). ***Paratypes***, same data as holotype except elevation, 99–145 m a.s.l.: ♀ (ICN 12359), ♂ (ICN 12360), ♂ (ICN 12361), ♀ (ICN 12362), ♂ (ICN 12363).

##### Etymology.

The specific epithet *pacifica* is a noun in feminine refers to the Colombian Pacific region, where the species is distributed.

##### Diagnosis.

Male of *Melloinapacifica* sp. nov. can be distinguished from other *Melloina* species by the relatively longer embolus (Fig. 17), ca. 2 × the tegulum length (< 2 × in *M.gracilis* (Schenkel, 1953), > 3 × in *M.santuario* Bertani, 2013), by the labium with numerous cuspules (112 vs. 60–80 in other known species), and by the tarsi II, III, IV ventrally cracked at midpoint (III and IV in *M.gracilis*, only IV in *M.santuario*). Additionally, differs from *M.santuario* by the number of spines on tarsi I and II (two rows of 8–10 spines vs. 4 or 5 in *M.santuario*, similar in *M.gracilis* 9–12). Female can be distinguished from other *Melloina* species by the straight, long, and wide spermathecae, without glandular area in the basal third (Fig. 19C), numerous maxillary cuspules (160 vs. 129/141 in *M.gracilis*, 82/90 in *M.santuario*, and 60 in *M.rickwesti* Raven, 1999), and labium with 98 cuspules (77 in *M.santuario*, ca. 90 in other species).

##### Distribution.

Known only from the type locality (Figs 1, 2).

##### Description.

**Figure 11.**
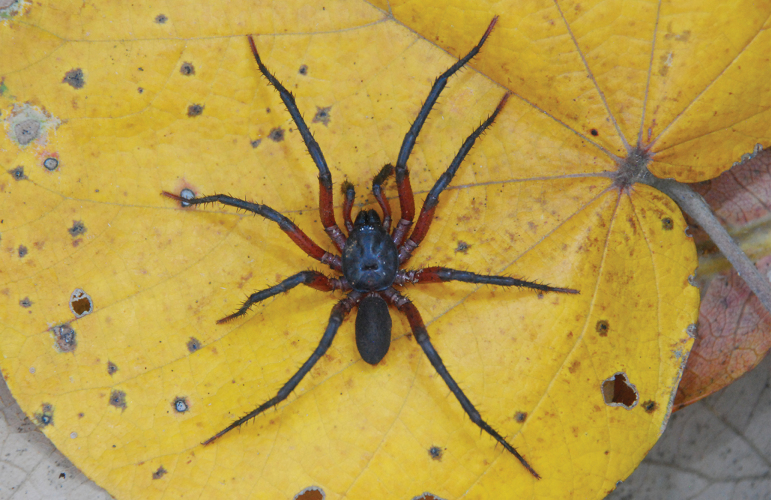
*Melloinapacifica* sp. nov., holotype male, habitus.

**Figure 12.**
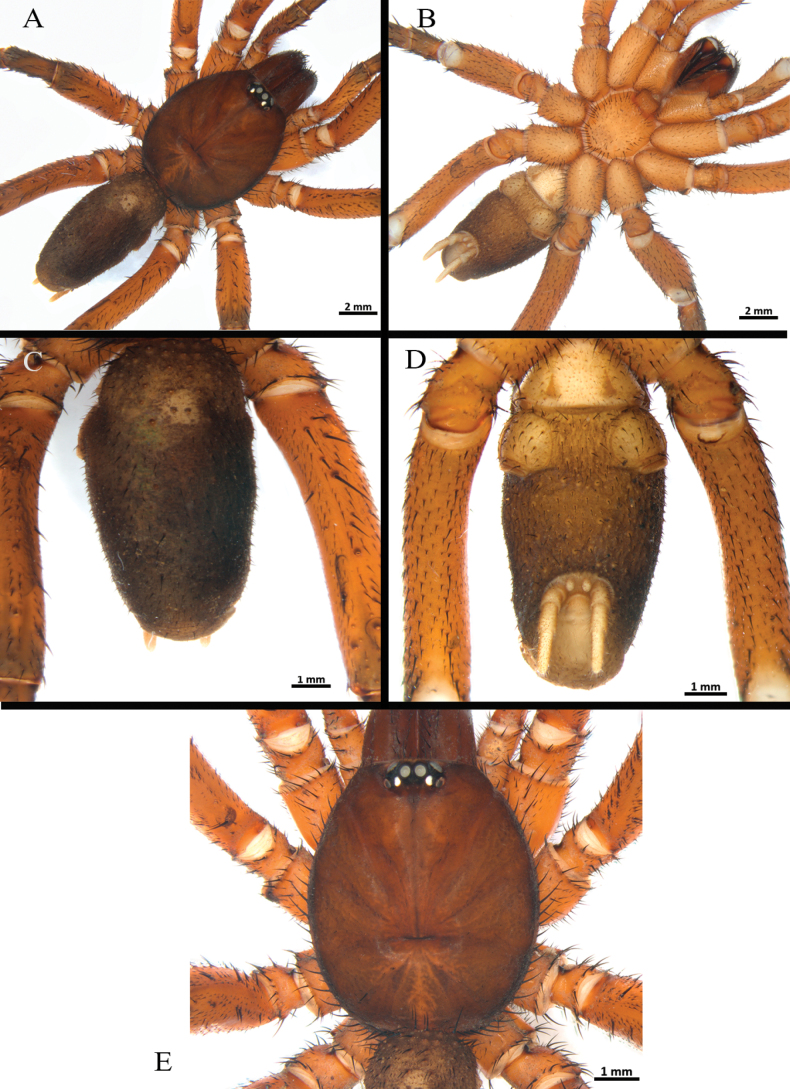
*Melloinapacifica* sp. nov., holotype male, habitus **A** dorsal view **B** ventral view **C, D** abdomen **C** dorsal view **D** ventral view **E** carapace.

**Figure 13.**
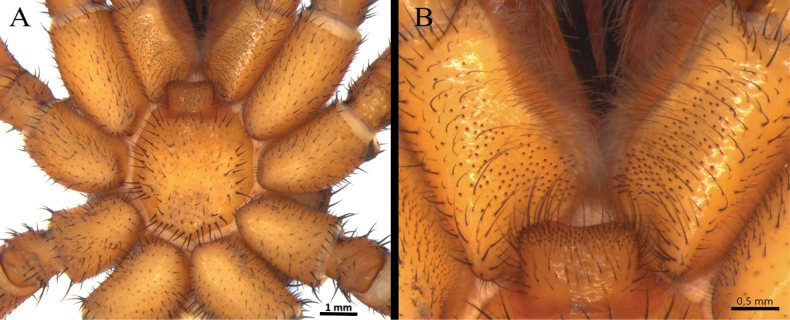
*Melloinapacifica* sp. nov., holotype male **A** sternum **B** labium and maxillae.

**Figure 14.**
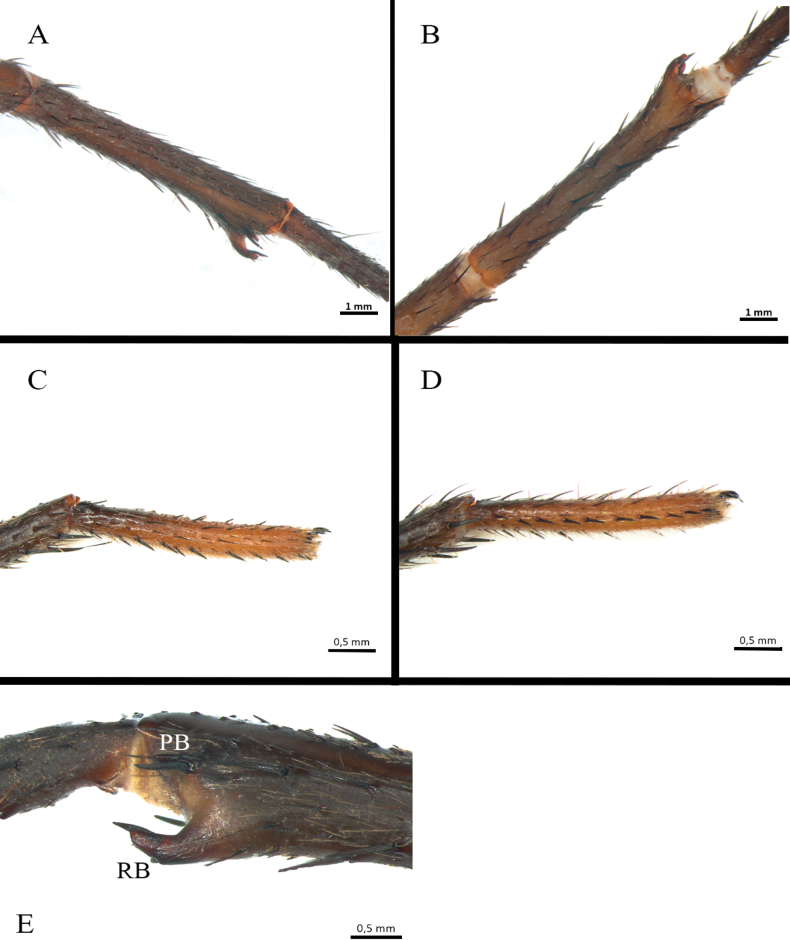
*Melloinapacifica* sp. nov., holotype male **A, B** tibia I **A** prolateral view **B** ventral view **C, D** tarsus I **C** prolateral view **D** retrolateral view **E** tibial apophysis on leg I. PB = prolateral branch, RB = retrolateral branch.

**Figure 15.**
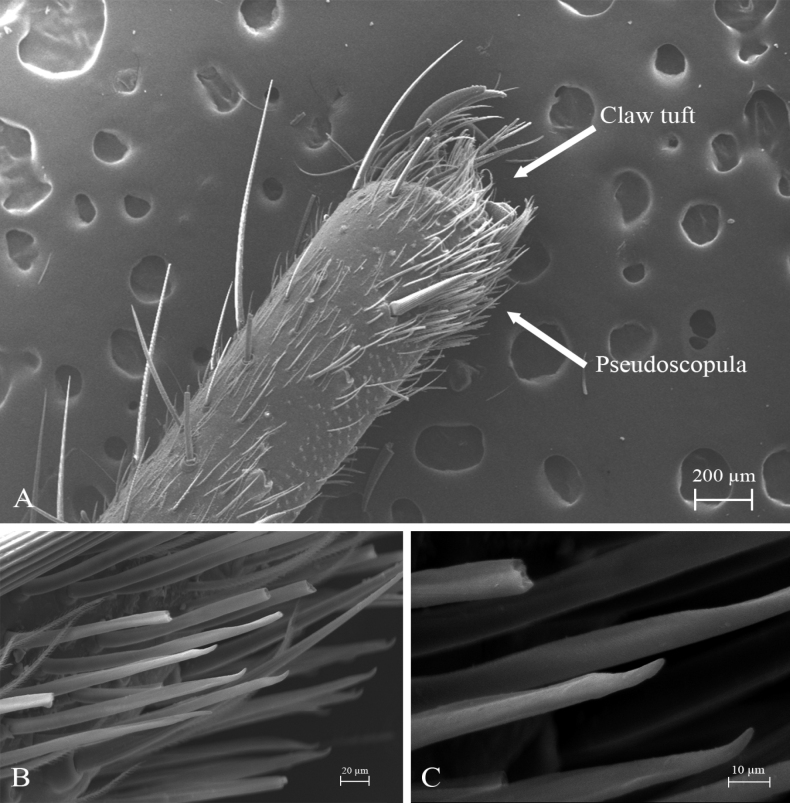
*Melloinapacifica* sp. nov., holotype male, tarsus I **A** lateral view **B** detail of pseudoscopula **C** detail of pseudoscopular setae.

**Figure 16.**
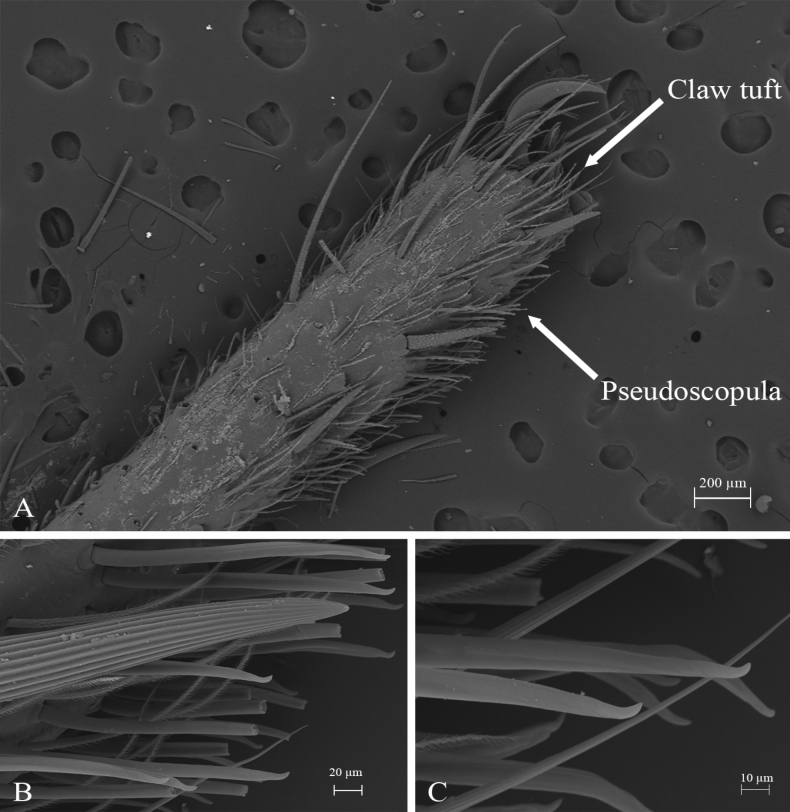
*Melloinapacifica* sp. nov., holotype male, tarsus II **A** lateral view **B** detail of pseudoscopula **C** detail of pseudoscopular setae.

**Figure 17.**
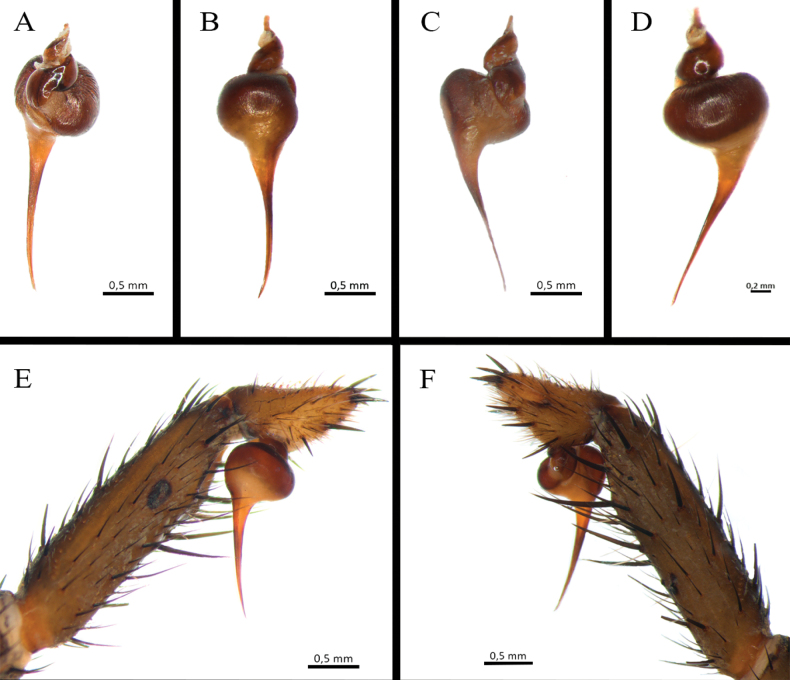
*Melloinapacifica* sp. nov., holotype male **A–D** copulatory bulb **A** ventral view **B** dorsal view **C** prolateral view **D** retrolateral view **E, F** palpal tibia and cymbium **E** retrolateral view **F** prolateral view.

**Male** (holotype) (Figs 11–17). Total length: 16.1. Chelicerae basal segment: length 2.1, width 1.4. Carapace: elongated, length 7.7, width 7.0; cephalic area slightly raised. Abdomen: ovoid, length 7.5, width 3.7. Spinnerets: PLS with three segments, total length 2.52 (basal 0.82, middle 0.75, apical digitiform 0.95); PMS with one segment, length 0.45. (Fig. 12).

Clypeus: absent. Ocular tubercle (Fig. 12E): ovoid, raised, forwardly directed, length 1.04, width 1.78. Anterior eye row procurved, posterior eye row slightly recurved. Eye diameters and interdistances: AME 0.39 (circular), ALE 0.46 (oval), PME 0.30 (oval), PLE 0.35 (oval), AME-AME 0.09, AME-ALE 0.19, PME-PME 0.72, PME-PLE 0.09, PLE-PLE 1.31, ALE-PLE 0.11, AME-PME 0.05. Thoracic fovea (Fig. 12E): transverse, straight, deep, width 2.19; 4.41 from the anterior edge of carapace. Chelicerae basal segment: spiniform setae on three rows on dorsal area and some dispersed on lateral areas, with 15 left/ 14 right well-developed teeth on each furrow promargin, and a group of ca. 43 small teeth near last three basal promargin teeth. Intercheliceral tumescence absent. Fang long. Maxillae (Fig. 13): longer than wide, trapezoidal, with ca. 111 left / 108 right cuspules, spaced, largely spread but more dense over ventral inner heel; the distal prolateral lobe conical and the proximal posterior angle projected. Labium (Fig. 13): sub-rectangular, length 0.77, width 1.15, with ca. 112 cuspules on anterior edge, evenly distributed. Labio-sternal junction: narrow in the midline with two oval sigillae touching and extended to the edge. Sternum (Fig. 13A): rounded, anterior edge with a semicircular area slightly raised (joined to labio-sternal groove), length 3.25, width 3.08, with three pairs of inconspicuous sigillae sclerotized. Sigillae: proximal pairs subcircular, submarginal; distal pair oval, marginal.

Legs pattern: IV>I>II>III. Lengths of legs and palpal segments on Table 3. Tarsal claws: STC long, with row of three or four small teeth, ITC absent on all legs. Claw tufts: weak, present in all tarsi (Figs 14C, D, 15, 16). Tarsal scopulae: pseudoscopula present, tarsi I and II distally with weak pseudoscopulae (Figs 14C, D, 15, 16), III with a few distal sparse setae, IV without scopulae. Metatarsal scopulae: absent in all metatarsi. Trichobothria: filiform of different sizes and clavate in all tarsi, I and II with ca. 22 filiform and ca. 12 clavate, III and IV with ca. 20 filiform and ca. eight clavate; filiform trichobothria also present in all metatarsi and tibia, including palpal tibia. Tarsi II, III, IV ventrally cracked at midpoint. Plumose setae on retrolateral face of femur IV: absent. Stridulatory bristles: absent. Body with strongly pilose setae. Book lung openings oval and sclerotized. Urticating setae: absent.

**Table 3.** *Melloinapacifica* sp. nov. Male holotype. Lengths of legs and palpal segments.

	I	II	III	IV	Palp
Femur	8.07	6.96	6.15	9.36	3.99
Patella	4.37	3.59	2.87	3.53	2.42
Tibia	7.31	5.44	4.37	7.43	3.46
Metatarsus	6.39	5.48	5.77	9.81	-
Tarsus	6.80	3.63	3.35	4.26	1.37
Total	32.94	25.1	22.51	34.39	11.24

Spination (proximal to distal). Femora: palp d 5-8-11, p 0-0-3, v 0-2-0, r 0-2-2; I d 6-4-10, p 0-0-3, v 3-4-10 (8 ap), r 0-0-5; II d 5-5-7, p 0-0-1, v 0-0-6 (5 ap), r 1-1-1; III d 5-5-4, p 3-2-2, v 1-3-10 (8 ap), r 1-2-3; IV d 5-2-4, p 0-0-1 ap, v 0-2-10 (8 ap), r 0-1-1. Patellae: palp d 9-7-8, p 0-1-4 ap, v 1-1-4 ap, r 2-3-2; I d 1-1-3, p 1-1-2, v 0-0-2 ap, r 1-1-2 ap; II d 3-2-5, p 1-2-5 (4 ap), v: 0-1-3 ap, r: 1-1-2; III d: 5-4-5, p: 2-2-2, v: 0-2-2 ap, r: 1-1-3 ap; IV d: 3-1-2, p: 0-1-2 ap, v 0-2-3 (2 ap), r 0-0-1 ap. Tibiae: palp d 2-5-5, p 4-2-4 (2 ap), v 5-6-7 (3 ap), r 2-3-8 (2 ap); I d 1-1-3, p 1-1-2, v 0-0-2 ap, r 1-1-2 ap; II d 2-1-1, p 3-2-3 (2 ap), v 4-4-4 (2 ap), r 2-2-0; III d 3-2-3, p 3-2-3 (1 ap), v 2-2-2 ap, r 3-3-3 ap; IV d 7-5-7, p 5-4-4 (1 ap), v 3-2-3 (2 ap), r 2-2-2 (1 ap). Metatarsi: I d 0, p 2-2-1 ap, v 6-4-6 (2 ap), r 1-1-1; II d 2-2-2, p 4-4-4 (1 ap), v 4-5-6 (3 ap), r 3-2-2 (1 ap); III d 4-4-3, p 2-2-2, v 4-4-5 (2 ap), r 2-3-3 (1 ap); IV d: 3-1-4, p: 2-2-2, v: 3-3-4 (2 ap), r: 3-2-2 (1 ap). Tarsi: cymbium p lobe 4, r lobe 10 (8 ap); I d 0, p 3-1-4 (1 ap), v 0, r 3-3-4 (1 ap); II d 1-1-1, p 3-3-4 (1 ap), v 0, r: 3-3-4 (1 ap); III d 3-2-3, p 3-3-4 (1 ap), v 0, r 3-3-4 (1 ap); IV d 3-4-3, p 2-3-3 (1 ap), v 2-1-2, r 2-3-3 (1 ap).

Palp (Fig. 17): palpal bulb pyriform, embolus long, thin, filiform at apex (Fig. 17A–D); cymbium of two dissimilar lobes, distally spinose (Fig. 17E, F); tibia with shallow distoventral groove. Tibial apophysis (Fig. 14E): composed of two unequal branches originating from common base; retrolateral branch longest, distally curved, with two large subapical spines, one internal and one external; prolateral branch straight, small, with a very large basal spine, longer than its length. Metatarsus I straight, when flexed it passes on the retrolateral side of the apophysis.

***Coloration*.** Living spider: carapace, palp, and legs reddish black; femora and tarsi red, distal femora, patellae, tibiae, and metatarsi black; abdomen dark (Fig. 11). In alcohol: carapace reddish brown; palp and legs brown; distal femora, patellae, tibiae, and metatarsi dark brown; abdomen greyish brown.

**Figure 18.**
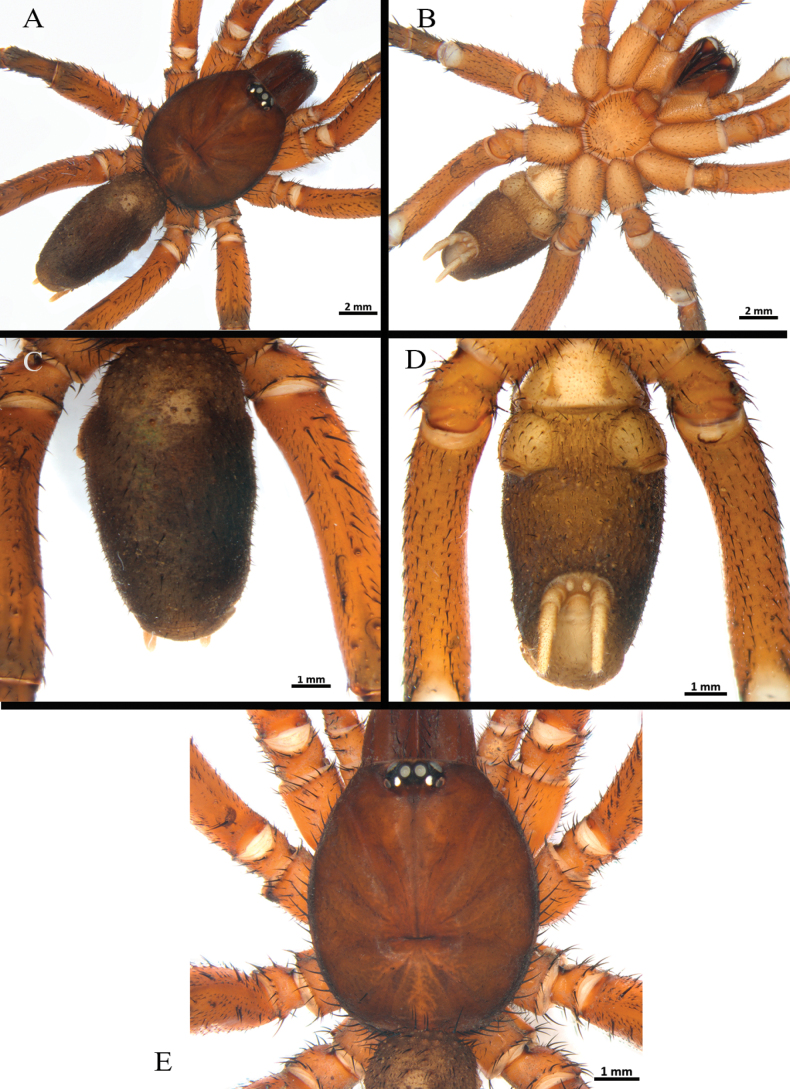
*Melloinapacifica* sp. nov., paratype female (ICN 12359), habitus **A** dorsal view **B** ventral view **C, D** abdomen **C** dorsal view **D** ventral view **E** carapace.

**Figure 19.**
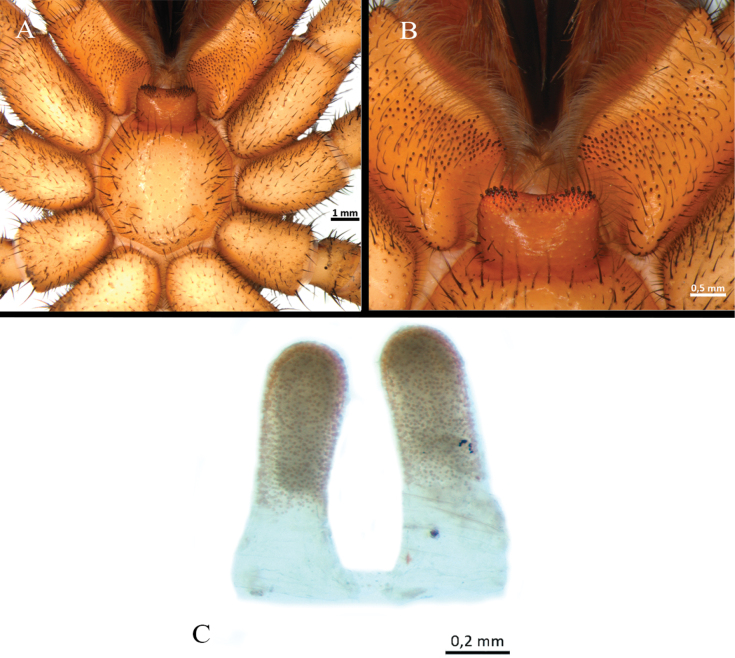
*Melloinapacifica* sp. nov., paratype female (ICN 12359) **A** sternum and coxae **B** labium and maxillae **C** spermathecae, ventral view.

**Female** (paratype - ICN 12359) (Figs 18, 19). Total length: 18.1. Chelicerae basal segment: length 2.0, width 1.7. Carapace: elongated, length 8.2, width 7.2; cephalic area slightly raised. Abdomen: ovoid, length 9.1, width 5.8. Spinnerets: PLS with three segments, total length 2.73 (basal 0.91, middle 0.75, apical digitiform 1.07); PMS with one segment, length 0.69. (Fig. 18).

Clypeus: absent. Ocular tubercle (Fig. 18E): ovoid, raised, forwardly directed, length 1.24, width 2.04. Anterior eye row procurved, posterior eye row slightly recurved. Eye sizes and interdistances: AME 0.32 (circular), ALE 0.48 (oval), PME 0.23 (circular), PLE 0.37 (oval), AME-AME 0.09, AME-ALE 0.14, PME-PME 0.8, PME-PLE 0.09, PLE-PLE 1.57, ALE-PLE 0.13, AME-PME 0.06. Thoracic fovea (Fig. 18E): transverse, straight, deep, width 2.51; 5.48 from the anterior edge of carapace. Chelicerae basal segment: spiniform setae on three rows on dorsal area and some dispersed on lateral areas, with 16 well-developed teeth on each furrow promargin, and group of ca. 43 small teeth near last three basal promargin teeth. Fang long. Maxillae (Fig. 19B): longer than wide, with the anterior prolateral lobe conical with ca. 160 cuspules, spaced, largely spread but more dense over ventral inner heel; the distal prolateral lobe conical and the proximal posterior angle projected. Labium (Fig. 19B): sub-rectangular, length 1.02, width 1.73, with ca. 98 cuspules on anterior edge distributed with higher density towards the lateral sides. Labio-sternal junction: narrow in the midline with two oval sigillae touching and extended to the edge. Sternum (Fig. 19A): rounded, anterior edge with a semicircular area slightly elevated (joined to labio-sternal groove), length 3.49, width 3.75, with three pairs of conspicuous sclerotized sigillae. Sigillae: proximal pair subcircular, submarginal; distal pairs oval, submarginal.

Legs pattern: IV>I>II>III. Lengths of legs and palpal segments on Table 4. Tarsal claws: STC long, with row of 2–4 small teeth, ITC absent on all legs. Claw tufts: weak, present in all tarsi. Tarsal scopulae: absent in all legs. Metatarsal scopulae: absent in all legs. Trichobothria: filiform of different sizes and clavate in all tarsi, I–IV with ca. 20 filiform, I–III with ca. ten clavate, IV with ca. seven clavate; filiform trichobothria also present in all metatarsi and tibia, including palpal tibia. Tarsus IV cracked at midpoint. Plumose setae on retrolateral face on femur IV: absent. Stridulatory bristles: absent. Body with strongly pilose setae. Book lung openings oval and sclerotized. Urticating setae: absent.

**Table 4.** *Melloinapacifica* sp. nov. Female paratype. Lengths of legs and palpal segments.

	I	II	III	IV	Palp
Femur	7.12	5.96	5.4	8.01	4.32
Patella	4.41	3.63	2.82	3.56	2.7
Tibia	6.17	4.54	3.82	6.84	3.1
Metatarsus	4.96	4.15	4.52	7.75	-
Tarsus	2.88	2.74	2.79	3.29	3.12
Total	25.54	21.02	19.35	29.45	13.24

Spination (proximal to distal). Femora: palp d 2-2-4, p 0-0-1, v 12-9-8, r 0; I d 3-2-3, p 0, v 3-3-5, r 0; II d 1-0-1, p 0, v 4-3-3, r 0; III d 1-2-1, p 1-1-1, v 1-2-3, r 0; IV d 1-1-1, p 0, v 1-2-6, r 0. Patellae: palp d 3-2-3, p 1-1-3 ap, v 0-0-3 ap, r 0-1-2; I d 0, p 0-1-1, v 0-2-2 ap, r 1-1-0; II d 0, p 0, v 0-2-2 ap, r 0; III d 0-1-0, p 0-1-1, v 0-1-2, r 0; IV d 0, p 0, v 0-2-3 (2 ap), r 0. Tibiae: palp d 5-5-3, p 0-1-2, v 6-5-3 ap, r 2-2-2; I d 4-6-4, p 2-1-2, v 3-4-2 ap, r 1-1-1; II d 2-1-1, p 1-1-2, v 4-5-4 (2 ap), r 1-1-1; III d 4-5-5, p 2-2-2, v 3-4-4 (3 ap), r 0-1-1; IV d 2-2-2, p 3-2-1 ap, v 4-5-5 (1 ap), r 1-1-2 (1 ap). Metatarsi: I d 0, p 1-1-0, v 5-5-6 (2 ap), r 0; II d 0, p 1-1-3, v 5-3-5 (2 ap), r 1-2-2 (1 ap); III d 2-3-4, p 2-2-2 (1 ap), v 4-3-3 (1 ap), r 2-2-2; IV d 4-4-4, p 2-2-1, v 4-3-4 ap, r 2-2-2. Tarsi: palp d 0, p 3-3-3, v 1-3-0, r 2-3-2; I d 0, p 3-3-4, v 0-0-2 ap, r 2-3-2; II d 0, p 3-3-3 (1 ap), v 0, r 3-3-3; III d 0, p 3-3-3, v 0, r 3-4-3; IV d 0, p 3-3-4 (1 ap), v 0-3-0, r 4-3-4 (1 ap).

Spermatheca (Fig. 19C): two seminal receptacles digitiform, straight, long, and wide, without glandular area in the basal third.

***Coloration*.** In alcohol: as described in the male.

##### Remarks.

*Melloinapacifica* sp. nov. is the first species of the genus described for Colombia, although it is known that *Melloina* is distributed in different ecosystems, including cave environments (Perafán 2017; Pérez-Miles et al. 2019). Males were captured actively walking at night and females were captured in shallow burrows, especially muddy ground. This record expands the geographical distribution of the genus, recorded previously only for Venezuela and Panama (WSC 2023). We confirmed the presence of pseudoscopulae in the anterior tarsi of males, as reported by Pérez-Miles et al. (2017). In the present work, *M.pacifica* sp. nov. is included within Theraphosidae according to phylogenetic analysis of Mori and Bertani (2020) and the preliminary results of Pérez-Miles et al. (2019). It should be noted, as mentioned by other authors, *Melloina* is a distinctive taxon since it is the only one with the exclusive combination of claw tufts and tarsal pseudoscopulae (only in males). Additionally, its taxonomic position is currently being widely debated (Raven 1985; Pérez-Miles et al. 2019; Mori and Bertani 2020; Goloboff-Szumik and Ríos-Tamayo 2022).

### ﻿Subfamily Schismatothelinae

**Genus *Euthycaelus* Simon, 1889**

#### ﻿ Euthycaelus cunampia sp. nov.

Taxon classificationAnimaliaAraneaeTheraphosidae

DA473CA4-679B-5F00-9995-F73851BA5DAE

https://zoobank.org/6003756C-D948-4BFC-A0D2-9895FC2B98DF

**Figure 20.**
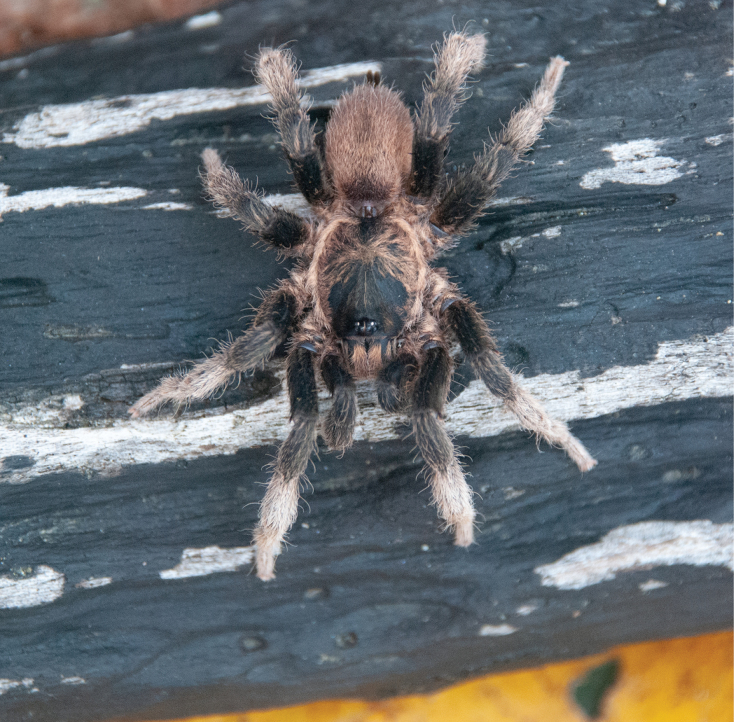
*Euthycaeluscunampia* sp. nov., holotype male, habitus.

**Figure 21.**
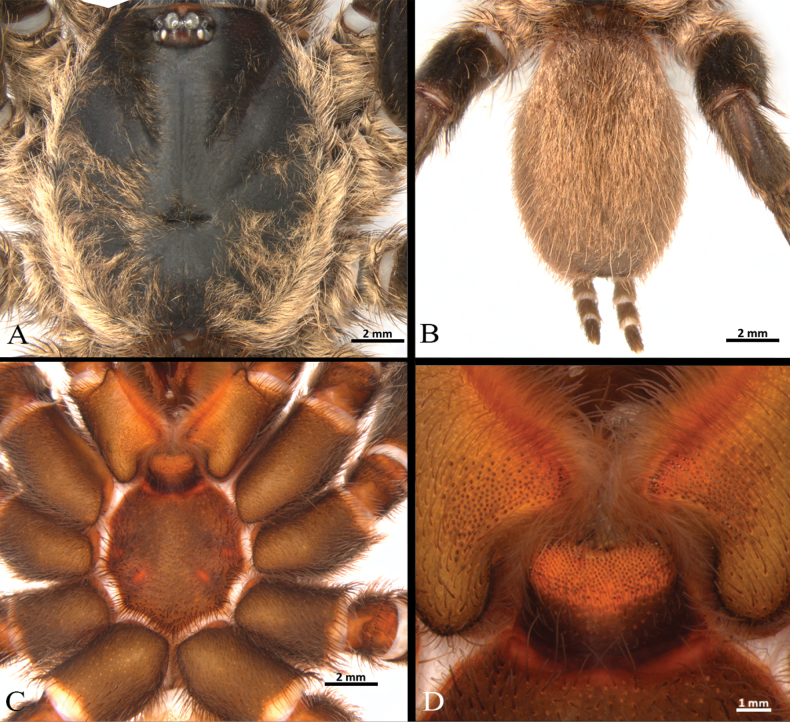
*Euthycaeluscunampia* sp. nov., holotype male **A** carapace **B** abdomen, dorsal view **C** sternum and coxae **D** labium.

**Figure 22.**
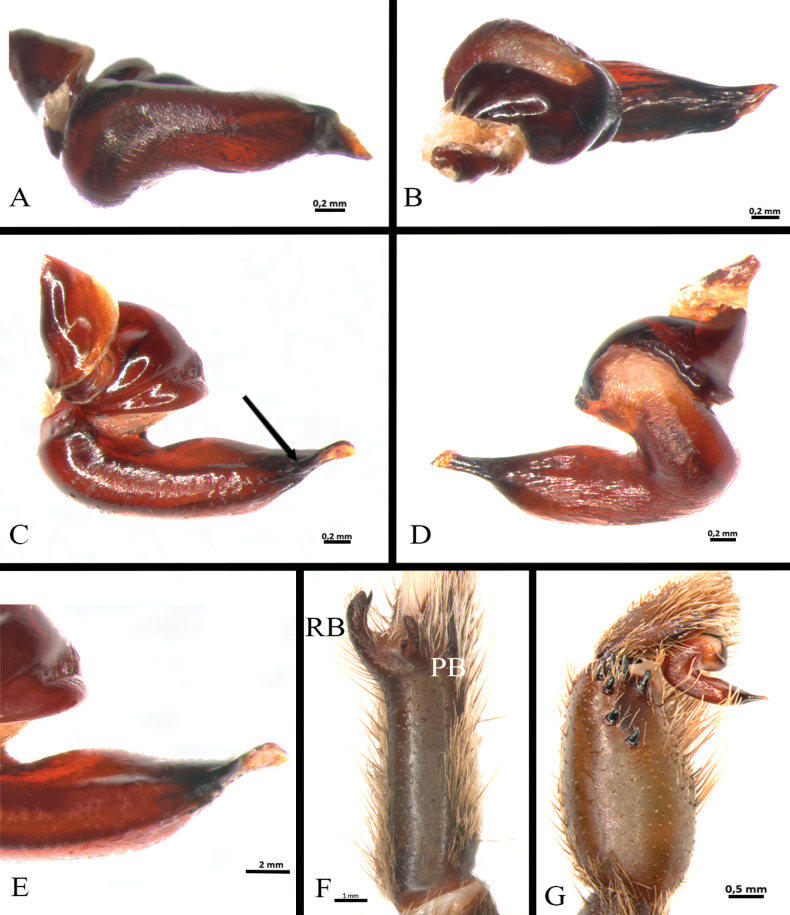
*Euthycaeluscunampia* sp. nov., holotype male **A–E** palpal bulb **A** ventral view **B** dorsal view **C** prolateral view **D** retrolateral view **E** detail of prolateral keels **F** tibial apophysis on leg I, prolateral view **G** palpal tibia. Arrow indicates prolateral keels. Abbreviations: PB = prolateral branch, RB = retrolateral branch.

Figs 20 , 21 , 22 ; Table 5

##### Type material.

***Holotype*** ♂: Colombia, Chocó, Bahía Solano, Jardín Botánico del Pacífico, 6.38, -77.40, elevation 124 m a.s.l., 10–25 February 2022, M. Echeverri, S. Gómez Torres and C. Perafán leg. (ICN 12364).

##### Etymology.

The specific epithet *cunampia* is a patronym in honor of the family name of Don José and Don Antonio, members of the Emberá indigenous community, from Mecana, Chocó. Mr. José and Mr. Antonio abandoned their hunting traditions for their community to become touristic and academic guides for the JBP. We want to pay tribute to their community and to the JBP with this recognition.

##### Diagnosis.

Males of *Euthycaeluscunampia* sp. nov. can be distinguished from all other *Euthycaelus* species by the following combination of morphological features: the shape of the palpal bulb (Fig. 22A–E), with subtegulum widely separated from tegulum, embolus elongated, broadened medially, and tip dorsoventrally flattened, with numerous prolateral keels near the apex and without denticles. Copulatory bulb of *E.cunampia* sp. nov. resembles males of *E.quinteroi* Gabriel & Sherwood, 2022, but differs by the wider embolus (Fig. 22C–E) with a curved inner edge (straight in *E.quinteroi*), and additionally by a higher number of maxillary cuspules (ca. 200 vs. 150) and labial cuspules (ca. 300 vs. 200). Additionally, males and females (alive) with carapace and legs black covered by light brown setae, and tibiae, metatarsi and tarsi covered with very light setae (Fig. 20).

##### Distribution.

Known only from the type locality (Figs 1, 2).

##### Description.

**Male** holotype (Figs 20–22). Total length: 20.78. Chelicerae basal segment: length 2.63, width 1.73. Carapace: elongated, length 10.45, width 8.51; cephalic area slightly raised. Abdomen: ovoid, length 9.66, width 6.41. Spinnerets: PLS with three segments, total length 4.66 (basal 1.74, middle 1.30, apical digitiform 1.62); PMS with one segment, length 1.0. (Fig. 21).

Clypeus: absent. Ocular tubercle (Fig. 21A): ovoid, slightly raised, length 1.1, width 1.98. Anterior eye row procurved, posterior eye row slightly recurved. Eyes sizes and interdistances: AME 0.60 (circular), ALE 0.50 (oval), PME 0.39 (subcircular), PLE 0.45 (oval), AME–AME 0.11, AME–ALE 0.13, ALE–ALE 1.22, PME–PME 0.98, PME–PLE 0.02, PLE–PLE 1.47, AME–PME 0.04, ALE–PLE 0.13. Thoracic fovea (Fig. 21A): slightly procurved, width 1.69; narrow, deep, 6.86 from the anterior edge of carapace. Chelicerae basal segment: with ten well-developed teeth on each furrow promargin, and a group of ca. 30 small teeth on proximal area of each furrow. Intercheliceral tumescence absent. Maxillae (Fig. 21D): longer than wide, with ca. 200 cuspules located at anterior inner corner. Labium (Fig. 21D): sub-rectangular, length 0.70, width 1.82, with ca. 300 cuspules on anterior edge, evenly distributed. Labio-sternal junction: narrow in the midline with two lateral mounds. Sternum (Fig. 21C): rounded, length 4.34; width 4.32, slightly raised on anterior middle area, with three pairs of oval sigillae heavily sclerotized. Sigillae: proximal pair circular, distal pairs oval; proximal pairs separated by its diameter from the edge, posterior pair separated by more than its diameter.

Legs pattern: IV>I>II>III. Lengths of legs and palpal segments on Table 5. Tarsal claws: STC with row of small teeth, ITC absent on all legs. Tarsal scopulae: tarsi I and II entire, III and IV divided by longitudinal band of conical setae. Metatarsal scopulae extent: I almost fully scopulated (90%), II distal 3/4 (75%), III more than distal half (60%), IV distal half (50%) sparsely scopulated proximally. Trichobothria: tarsi with two rows of clavate trichobothria, each with ca. 25, interspersed with filiform trichobothria of different sizes. Tarsus IV slightly cracked at midpoint. Femur III: slightly incrassate. Plumose setae on retrolateral face of femur IV: absent. Stridulatory bristles: absent. Urticating setae: absent.

**Table 5.** *Euthycaeluscunampia* sp. nov. Male holotype. Lengths of legs and palpal segments.

	I	II	III	IV	Palp
Femur	7.83	6.81	6.29	8.40	5.41
Patella	4.97	4.13	3.41	4.06	3.0
Tibia	6.06	4.87	3.83	6.50	4.43
Metatarsus	6.02	5.21	5.40	8.53	-
Tarsus	3.96	3.31	3.05	3.79	2.34
Total	28.89	24.33	21.98	31.28	15.18

Spination (proximal to distal). Cymbium and tarsi without spines. Femora: palp d 0-0-1p; I d 0-0-1p; II d 0-0-1p; III d 0-0-2p-r; IV d 0-0-1r. Patella: I–II, IV and palp 0; III r 0-0-1d. Tibiae: palp p 0-0-1d, r 0-0-7; I v 0-0-1, p 0-0-1; II v 0-0-2 (ap), p 0-0-1; III d 1-0-2, v 1-2-2 (ap); IV d 2-1-2, v 2-2-3 (ap). Metatarsi: I v 1-0-3 (ap); II v 1-0-3 (ap); III d 1-2-2, v 1-2-3 (ap); IV d 1-2-2, v 1-2-4 (3 ap).

Palp (Fig. 22): palpal bulb elongated, wide, subtegulum widely separated from tegulum, tegulum strongly curved, with elongate embolus tip dorsoventrally relatively flattened, and with numerous prolateroventral keels (Fig. 22A–E). Palpal tibia (Fig. 22G): heavily incrassate with seven distal megaspines, arranged in two rows. Palpal cymbium: two asymmetrical lobes, the retrolateral larger and the prolateral elongated and laterally flattened. Tibia I: with paired tibial apophysis (Fig. 22F), RB longer than PB; PB with one short and developed megaspine with a pointed apex, RB with one short subapical and developed megaspine with a pointed apex (Fig. 22F). Metatarsus I: straight, otherwise unmodified, when flexes it passes on the retrolateral side of the RB.

***Coloration*.** Living spider: carapace black, covered by brown setae; palp and legs black, femora and patellae darker; tibiae, metatarsi and tarsi covered by very light setae; abdomen brown (Fig. 20).

**Female.** Unknown.

##### Remarks.

*Euthycaeluscunampia* sp. nov. represents the first published record of the genus and subfamily Schismatothelinae outside the Andean Region and the Eastern Cordillera for Colombia. This species constitutes the northernmost and westernmost record of the genus and subfamily for the country. Previously, the genus had a characteristic cis-Andean distribution over the Eastern Cordillera of Colombia and the Cordillera de Mérida in Venezuela (Valencia-Cuéllar et al. 2019). The geographic range of the genus *Euthycaelus* was recently extended with the publication of *E.quinteroi* from Panama, which is distributed in the same biogeographical region as *E.cunampia* sp. nov., in the Darien Gap, Chocó Biogeographical Region. This record extended the distribution of the genus to Central America. These latest records disrupt the distribution of the *Euthycaelus*, now being interpreted as a disjunct distribution, offering new evidence of historical connections between the Pacific humid forests and the Andean forests of the Eastern Cordillera.

### ﻿Subfamily Theraphosinae

**Genus *Neischnocolus* Petrunkevitch, 1925**

#### ﻿ Neischnocolus mecana sp. nov.

Taxon classificationAnimaliaAraneaeTheraphosidae

895FF42D-D5C8-5E6C-877F-9836DC85823F

https://zoobank.org/89DF166B-65AA-4F12-A1AA-3FFACD5A570F

**Figure 23.**
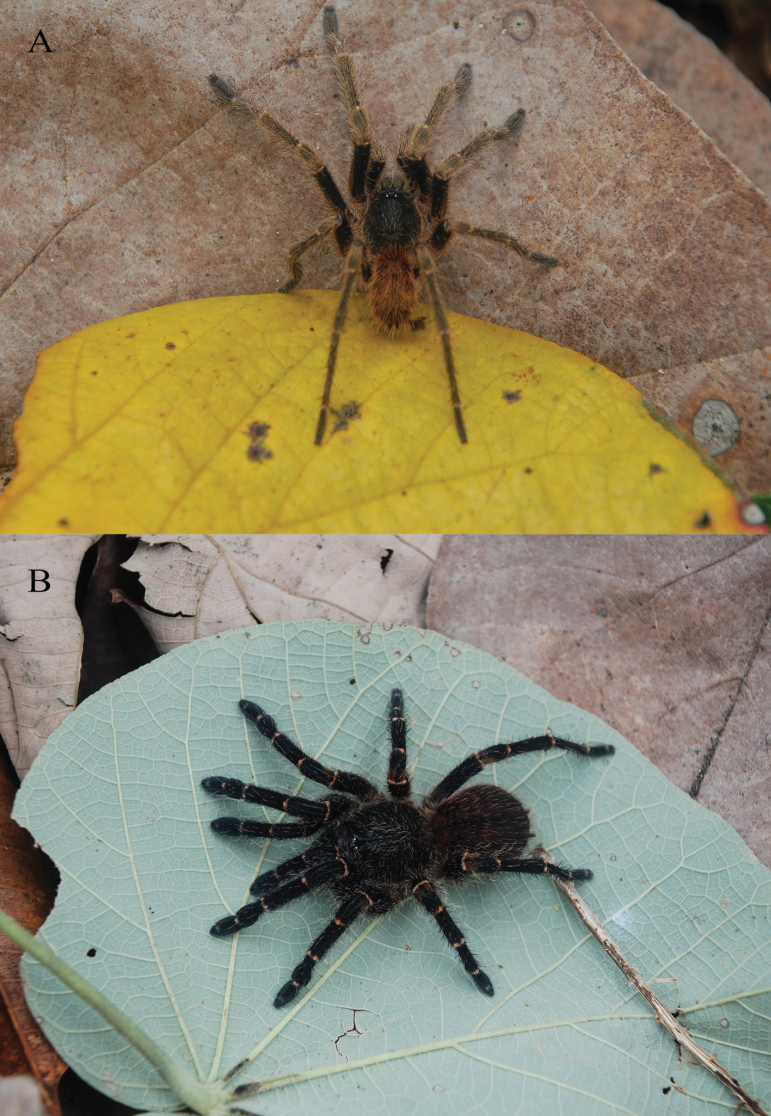
*Neischnocolusmecana* sp. nov., habitus **A** holotype male **B** paratype female.

**Figure 24.**
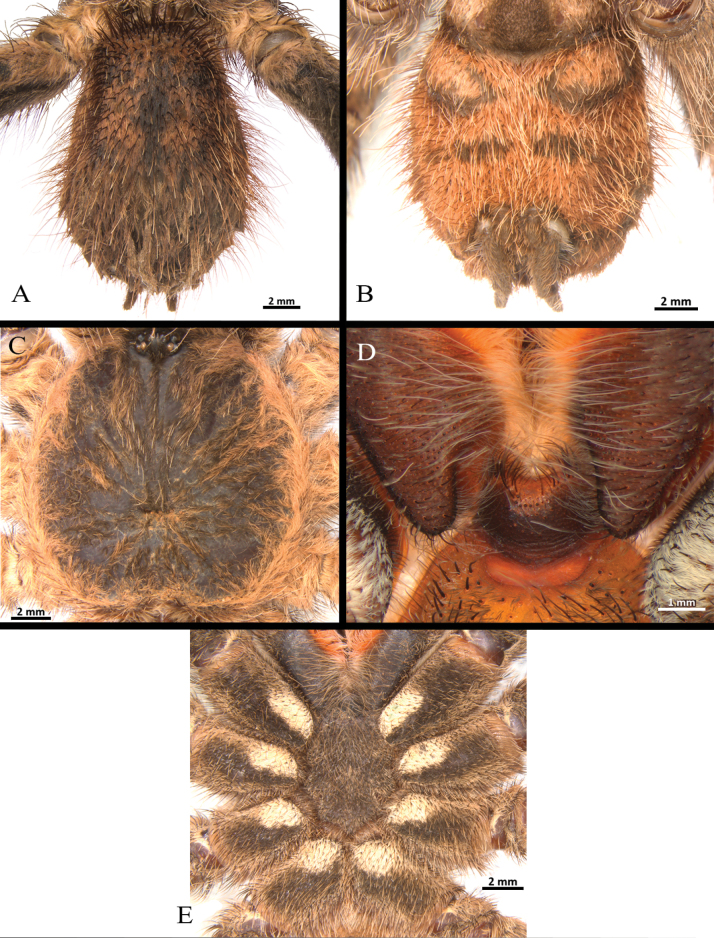
*Neischnocolusmecana* sp. nov., holotype male **A, B** abdomen **A** dorsal view **B** ventral view **C** carapace **D** labium and maxillae **E** sternum and coxae.

**Figure 25.**
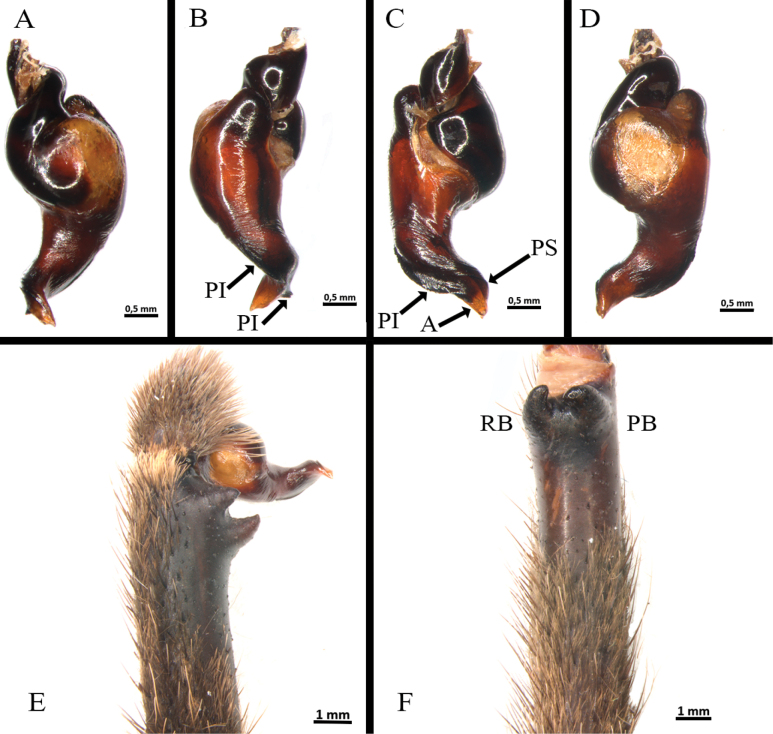
*Neischnocolusmecana* sp. nov., holotype male **A–D** copulatory bulb **A** ventral view **B** dorsal view **C** prolateral view **D** retrolateral view **E** palpal tibia **F** apophysis tibial on leg I, prolateroventral view. Abbreviations: A = apical keel, PB = prolateral branch, PI = prolateral inferior keel, PS = prolateral superior keel, RB = retrolateral branch.

**Figure 26.**
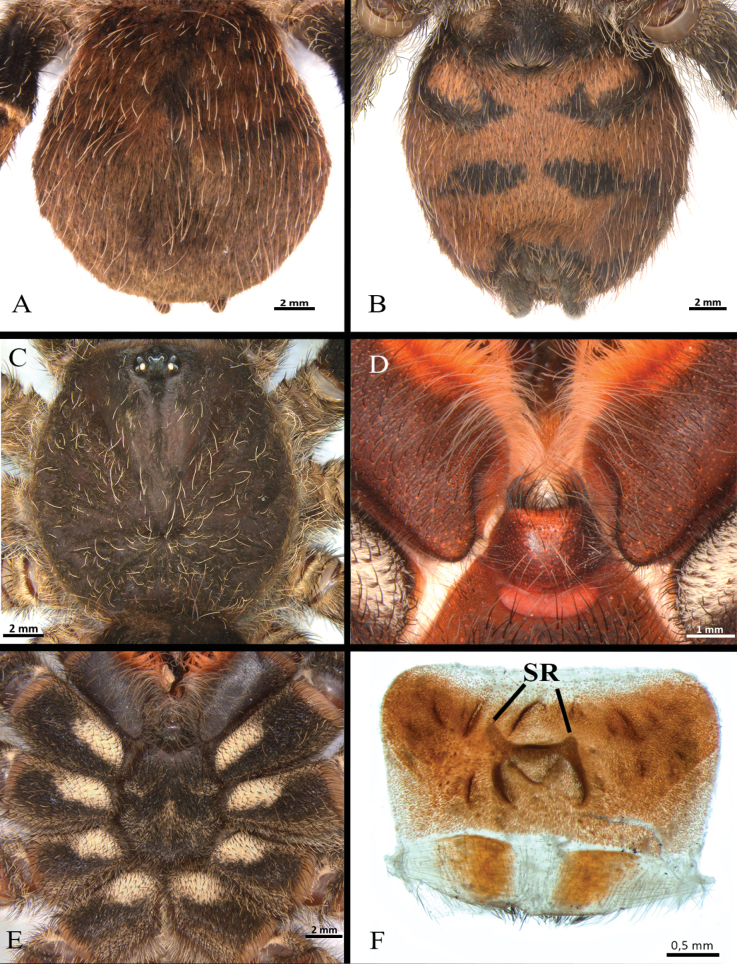
*Neischnocolusmecana* sp. nov., paratype female **A, B** abdomen **A** dorsal view **B** ventral view **C** carapace **D** labium and maxillae **E** sternum and coxae **F** spermathecae, ventral view. Abbreviation: SR = seminal receptacles.

Figs 23 , 24 , 25 , 26 ; Tables 6 , 7

##### Type material.

***Holotype*** ♂: Colombia, Chocó, Bahía Solano, Jardín Botánico del Pacífico, 6.38, -77.40, elevation, 28 m a.s.l., 10–25 February 2022, M. Echeverri, S. Gómez Torres and C. Perafán leg. (ICN 12365). ***Paratype*** ♀: same data as holotype (ICN 12366).

##### Etymology.

The specific epithet *mecana* is a noun in apposition related to one of the townships of the municipality of Bahía Solano, where the JBP is located. The name of this small town is due to the fact that it is located on the Mecana riverside, with crystalline waters and abundant biodiversity. The JBP promotes the conservation, research, and recovery of the native biodiversity of this region. We would like to pay tribute to its community and the JBP with this recognition.

##### Diagnosis.

Male of *Neischnocolusmecana* sp. nov. can be distinguished from the other *Neischnocolus* species by the following combination of morphological characters: shape of the palpal bulb piriform, with the tip of the embolus continuing the palpal organ axis (not perpendicular), well-developed prolateral (PS and PI) and apical (A) keels with non-serrated edge, PI discontinuous, absence of retrolateral keel (R), and without granulation or microspikes on embolus or tegulum (Fig. 25A–D); palpal tibia with two distal retrolateral processes (Fig. 25F). Female of *Neischnocolusmecana* sp. nov. differs from other *Neischnocolus* species in the spermatheca morphology consisting of a glandular and slightly sclerotized trapezoidal back-plate with small transverse keels, with two small asymmetrical tubiform seminal receptacles located on a small projected central portion of the atrium (Fig. 26F). Additionally, male and female have ventral coloration pattern (Figs 24B, E, 26B, E), and female has all body black color (Fig. 23B) (brown or reddish brown in the other species).

##### Distribution.

Known only from the type locality (Figs 1, 2).

##### Description.

**Male** holotype (Figs 23A, 24, 25). Total length 30.5. Carapace: length 14.7, width 14.2; cephalic area slightly raised. Abdomen: length 14.7, width 10.0. Spinnerets: PLS with three segments: total length 5.88 (basal 1.78, middle 1.70, apical digitiform 2.40); PMS with one segment, length 1.14. (Fig. 24).

Clypeus: absent. Ocular tubercle: ovoid, length 1.14, width 2.34; elevated, forwardly directed. Anterior eye row slightly procurved, posterior eye row slightly recurved. Eye diameters and interdistances: AME 0.54 (circular), ALE 0.68 (oval), PME 0.40 (oval), PLE 0.55 (oval), AME-AME 0.30, AME-ALE 0.18, PME-PME 1.11, PME-PLE 0.19, PLE-PLE 1.80, ALE-PLE 0.24, AME-PME 0.16. Thoracic fovea: transverse, width 3.25; slightly procurved, deep, 8.75 from the anterior edge of carapace. Chelicerae basal segment: length 4.2, width 3.1; with 11 well-developed teeth on each furrow promargin, and a group of ca. 15 small teeth near last three basal promargin teeth. Maxillae (Fig. 24D): longer than wide, with the anterior prolateral lobe conical; with ca. 45 left / 34 right cuspules covering ca. 75% of the proximal edge. Labium (Fig. 24D): sub-rectangular, length 1.43, width 2.64, with ten cuspules on anterior edge. Labio-sternal junction: broad in the midline with two sigillae joined. Sternum (Fig. 24E): oval with raised posterior angle, length 6.19, width 5.15, with three pairs of sigillae heavily sclerotized. Sigillae: proximal pair circular, distal pairs oval; proximal pair separated by more than its diameter from the edge, middle pair separated by less than its diameter, and posterior pair separated by its diameter.

Legs pattern: IV>I>II>III. Lengths of legs and palpal segments on Table 6. Trichobothria: filiform and clavate; all tarsi with two irregular longitudinal rows of short claviform trichobothria. Tarsal claws: STC with a row of six or seven small teeth, ITC absent. Scopulae: All tarsi 100% scopulate. Tarsal scopulae: tarsi I–III with scopula entire, tarsus IV divided by a wide band of longer conical setae; all tarsal scopulae with distal rhomboidal group of longer conical setae. Metatarsal scopulae extent: metatarsus I 60%, II 50%, III 15%, IV ascopulate. Plumose setae on retrolateral face of femur IV absent. Stridulatory bristles absent.

**Table 6.** *Neischnocolusmecana* sp. nov. Male holotype. Lengths of legs and palpal segments.

	I	II	III	IV	Palp
Femur	14.95	14.16	13.02	16.18	9.45
Patella	7.75	7.37	6.15	6.55	5.51
Tibia	12.31	10.90	10.20	13.56	7.43
Metatarsus	11.02	10.91	12.01	18.56	-
Tarsus	6.87	6.06	4.89	6.32	3.51
Total	53	49.4	46.17	61.17	25.9

Urticating setae: types I urticating setae present, subtype I_c_ (Kaderka et al. 2019) and I modified (Pérez-Miles et al. 2008) / subtype I_d_ (Kaderka et al. 2019), located in a dorsoposterior abdominal patch.

Spination: All femora, patellae, and tarsi 0. Legs I–II and palp 0. Tibiae: I–II 0; III d 0-0-1, v 0-1-2 ap, p 1-1-0, r 0-1-0; IV d 0-1-0, v 0-1-2 ap, p 0-1-1, r 0-0-1. Metatarsi: I–II 0; III d 0, v 0-2-4 (3 ap), p 1-1-1, r 0-1-1; IV d 0-0-1, v 2-3-4 (3 ap), p 1-1-1, r 0-1-1.

Palp (Fig. 25A–E): palpal bulb pyriform shape (Fig. 25A–D), embolus stout with the tip continuing the palpal organ axis, with two well-developed prolateral keels (PS and PI) and apical keel (A) present, PS discontinuous; without granulation on embolus or tegulum; tegular apophysis developed. Palpal tibia with two distinct subconical distal processes on retrolateral surface (Fig. 25E). Cymbium with two unequal lobes. Tibial apophysis (Fig. 25F): composed of two similar proventral branches, convergent, fused in their base. Flexion of metatarsus I retrolateral with respect to tibial apophysis.

***Coloration*.** Living spiders: body color brown, carapace and femora dark brown, legs brown, and abdomen reddish brown. Ventral abdomen with patterns of dark spots (Fig. 24B) and coxae with anterior-proximal white spots (Fig. 24E). In alcohol: reddish brown.

**Female** paratype (Figs 22B, 25). Total length 33.4. Carapace: length 15.0, width 14.5; cephalic area raised. Abdomen: length 17.6, width 14.0. Spinnerets: PLS with three segments, total length 9.21 (basal 3.14, middle 2.58, apical digitiform 3.49); PMS with one segment, length 2.05. (Fig. 26).

Clypeus: absent. Ocular tubercle: ovoid, length 1.34, width 2.62; elevated, forwardly directed. Anterior eye row slightly procurved, posterior eye row slightly recurved. Eye sizes and interdistances: AME 0.53 (circular), ALE 0.70 (oval), PME 0.41 (oval), PLE 0.50 (oval), AME-AME 0.34, AME-ALE 0.30, PME-PME 1.27, PME-PLE 0.26, PLE-PLE 2.00, ALE-PLE 0.36, AME-PME 0.21. Thoracic fovea: transverse, width 3.50; slightly procurved, deep, 9.11 from the anterior edge of carapace. Chelicerae basal segment: length 5.1, width 3.6; with 11 well-developed teeth on each furrow promargin, and a group of ca. 12 small teeth near last three basal promargin teeth. Maxillae (Fig. 26D): longer than wide, with the anterior prolateral lobe conical; with ca. 50 cuspules covering ca. 70% of the proximal edge. Labium (Fig. 26D): sub-quadrate, length 1.17, width 2.14, with 22 cuspules on anterior edge. Labio-sternal junction: broad in the midline with two sigillae joined. Sternum (Fig. 26E): rounded with raised posterior angle, length 6.19, width 6.18, with three pairs of sigillae heavily sclerotized. Sigillae: oval, proximal pair separated by more than its diameter from the edge, posterior pairs separated by its diameter.

Legs pattern: IV>I>II>III. Lengths of legs and palpal segments on Table 7. Trichobothria: filiform and clavate; all tarsi with two irregular longitudinal rows of short claviform trichobothria. Tarsal claws: STC with a row of five or six small teeth, ITC absent. Scopulae: All tarsi 100% scopulate. Tarsal scopulae: tarsi I–III scopula entire, tarsus IV divided by a wide band of longer conical setae; all tarsal scopula with distal rhomboidal group of longer conical setae. Metatarsal scopulae extent: metatarsus I 90%, II 50%, III 30%, IV ascopulate. Plumose setae on retrolateral face of femur IV absent. Stridulatory bristles absent.

**Table 7.** *Neischnocolusmecana* sp. nov. Female paratype. Lengths of legs and palpal segments.

	I	II	III	IV	Palp
Femur	11.24	10.50	8.79	12.07	9.16
Patella	7.53	6.34	5.54	6.57	5.90
Tibia	8.04	6.54	6.30	8.80	6.14
Metatarsus	6.20	5.83	7.69	12.07	-
Tarsus	4.00	3.34	3.82	4.17	5.27
Total	37.01	32.55	32.14	43.68	26.47

Urticating setae: types I urticating setae present, subtype I_c_ (Kaderka et al. 2019) and I modified (Pérez-Miles et al. 2008) / subtype I_d_ (Kaderka et al. 2019), located in a dorsoposterior abdominal patch; with a clearly higher proportion of urticating setae subtype I_d_ (100:1).

Spination: All femora, patellae, and tarsi 0. Tibiae: palp 0-0-2 ap; I d 0, v 0-0-2 ap, p 0, r 0; II d 0, v 0-0-3 ap, p 0-1-0, r 0; III d 1-0-0, v 0-1-3 ap, p 1-1-0, r 0-1-1; IV d 0, v 0-1-3 ap, p 1-1-0, r 1-1-1. Metatarsi: I 0; II d 0, v 0-1-2 ap, p 0-1-0, r 0; III d 0-1-1, v 0-3-2 ap, p 0-0-1 ap, r 0-0-1 ap; IV d 0-0-1, v 3-4-5 (3 ap), p 1-2-0, r 0-1-1.

Spermathecae (Fig. 26F): consisting of a glandular and slightly sclerotized trapezoidal back-plate with small transverse keels, with two small asymmetrical tubiform seminal receptacles located on a short projected central portion of the atrium.

***Coloration*.** Living spiders: carapace, abdomen, and legs black, legs with light-colored stripes at the joints. Ventral abdomen with patterns of dark spots (Fig. 26B) and coxae with anterior-proximal white spots (Fig. 26E). In alcohol: reddish brown, darker than male.

##### Remarks.

*Neischnocolusmecana* sp. nov. it is the fourth species of the genus described for Colombia and it is the first record of *Neischnocolus* for the Chocó biogeographic region, as well as the first record for the Colombian Pacific. With this description, the known geographic range of the genus is extended. Currently, *Neischnocolus* is distributed in Colombia in the Andean, Amazonian, and Pacific regions. It is known that *Neischnocolus* is widely distributed in the Colombian territory, with a very extensive geographical and altitudinal range, and that most of its diversity has not yet been described (Perafán 2017).

## Supplementary Material

XML Treatment for Ummidia solana

XML Treatment for Melloina pacifica

XML Treatment for Euthycaelus cunampia

XML Treatment for Neischnocolus mecana

## ﻿Additional information

### Author ORCIDs

Nicolás Pinel https://orcid.org/0000-0003-1304-3096

### Data availability

All of the data that support the findings of this study are available in the main text or Supplementary Information.
